# Two-Photon Imaging of Cortical Surface Microvessels Reveals a Robust Redistribution in Blood Flow after Vascular Occlusion

**DOI:** 10.1371/journal.pbio.0040022

**Published:** 2006-01-03

**Authors:** Chris B Schaffer, Beth Friedman, Nozomi Nishimura, Lee F Schroeder, Philbert S Tsai, Ford F Ebner, Patrick D Lyden, David Kleinfeld

**Affiliations:** **1**Department of Physics, University of California San Diego, La Jolla, California, United States of America; **2**Center for Theoretical Biological Physics, University of California San Diego, La Jolla, California, United States of America; **3**Department of Neurosciences, University of California San Diego, La Jolla, California, United States of America; **4**Department of Neurology, Veterans Affairs Medical Center, San Diego, California, United States of America; **5**Graduate Program in Neurosciences, University of California San Diego, La Jolla, California, United States of America; **6**Department of Psychology, Vanderbilt University, Nashville, Tennessee, United States of America; Washington University School of MedicineUnited States of America

## Abstract

A highly interconnected network of arterioles overlies mammalian cortex to route blood to the cortical mantle. Here we test if this angioarchitecture can ensure that the supply of blood is redistributed after vascular occlusion. We use rodent parietal cortex as a model system and image the flow of red blood cells in individual microvessels. Changes in flow are quantified in response to photothrombotic occlusions to individual pial arterioles as well as to physical occlusions of the middle cerebral artery (MCA), the primary source of blood to this network. We observe that perfusion is rapidly reestablished at the first branch downstream from a photothrombotic occlusion through a reversal in flow in one vessel. More distal downstream arterioles also show reversals in flow. Further, occlusion of the MCA leads to reversals in flow through approximately half of the downstream but distant arterioles. Thus the cortical arteriolar network supports collateral flow that may mitigate the effects of vessel obstruction, as may occur secondary to neurovascular pathology.

## Abstract 2

The authors quantify changes in blood flow in the pial arteriolar network of rodent cortex following targeted occlusions to individual vessels.

## Introduction

Normal brain function requires adequate levels of blood flow to ensure the delivery of oxygen and nutrients to cells and to facilitate the removal of metabolites and heat. The vasculature that supplies and regulates this flow is comprised of a succession of feeder vessels and networks with extensive redundant connections [1,2]. At the level of the supply to the brain, a pair of “communicating” arteries connects the carotid arteries to form the Circle of Willis. This loop is known to ensure a substantial level of fault tolerance to an occlusion of one of the member vessels [3]. At the level of cerebral cortex, the branches of each cerebral artery form the artery's own network of communicating arterioles on the surface of its cortical territory [4]. This network, in turn, gives rise to arterioles that plunge into the cortex and branch into the capillary bed. Each of the surface communicating networks is highly interconnected and, in addition, connects with the communicating networks of neighboring cerebral arteries through pial arteries called leptomeningeal anastomoses [5]. In analogy with the large-scale redundancy afforded by the Circle of Willis, the communicating network has been hypothesized to provide a robust source of collateral flow in the event of an occlusion of a microvessel [5–8]. Previous efforts to test this hypothesis, as well as the more general issue of the relationship between network topology and compensatory flow after an occlusion [3,9,10], have been hampered by a lack of methodology in which small vascular occlusions can be precisely targeted in time and space and in which flow can be quantified throughout multiple neighboring branches in a network.

Here we address the issue of flow redistribution in a network of cortical communicating arterioles in rat parietal cortex that is supplied primarily by the middle cerebral artery (MCA). Our approach makes use of optical-based technologies. Two-photon laser scanning microscopy (TPLSM) [11,12] in conjunction with labeling of the blood plasma with fluorescein-dextran is used to form maps of the angioarchitecture as well as quantify the transport of individual red blood cells (RBCs) [13–15]. Occlusions are performed in two complementary ways. In the first method, occlusions to individual targeted microvessels at the pial surface are achieved through the introduction of a photosensitizer to the blood stream and the subsequent irradiation of the microvessel with focused laser light. This technique requires, on the one hand, suprathreshold illumination of a surface vessel to form a clot and, on the other hand, subthreshold illumination of deep and lateral vessels to avoid undesired clots. Fulfillment of these conditions represents an extension of the conventional application of the photothrombotic clot method [16–20] to allow for examination of dynamic flow changes in surface communicating arterioles after focal clot formation in a single surface arteriole. In the second method, an overall decrease in perfusion through the arterial source to parietal cortex is achieved by threading a filament along the carotid artery to block the base of the MCA [21]. This decrease necessarily leads to a change in the overall pressure balance throughout parietal cortex [22].

## Results

### Mapping and Perturbing Blood Flow in Communicating Arteriole Networks

**Figure 1:**
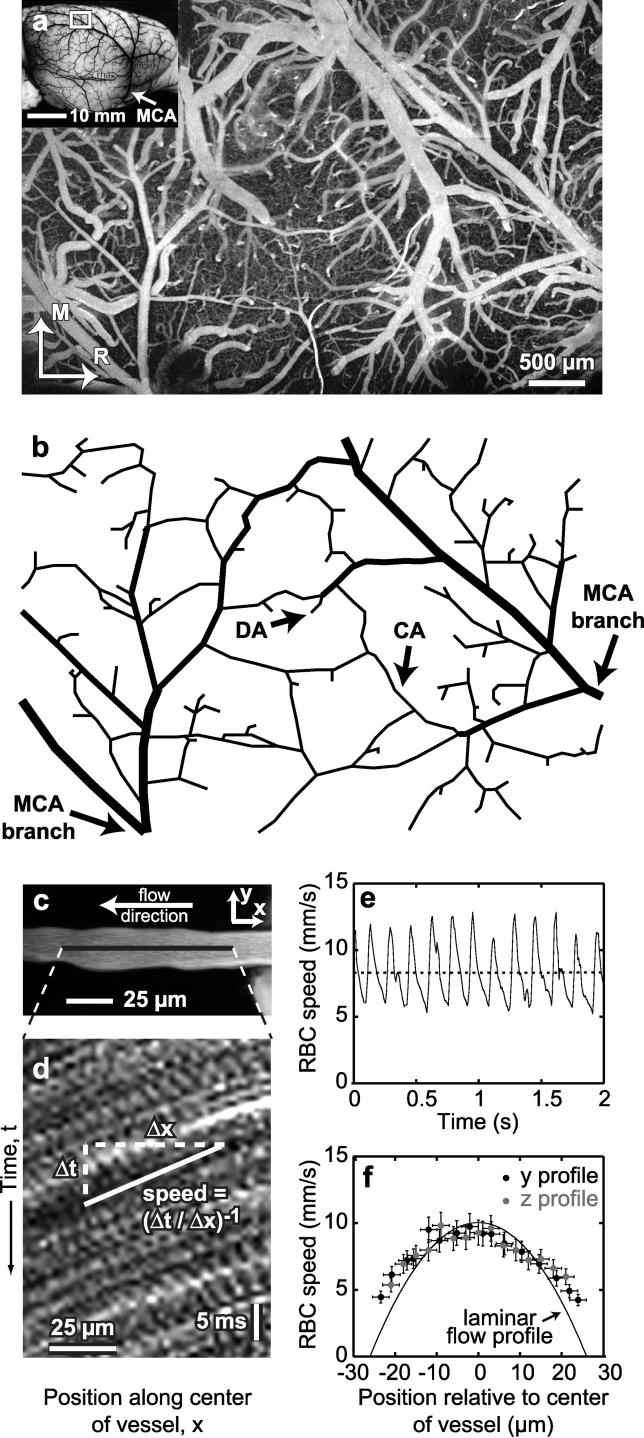
TPLSM of Fluorescently Labeled Cortical Vasculature In Vivo (A) Low-magnification TPLSM image of fluorescently labeled brain vasculature in rat parietal cortex. The axes indicate the rostral (R) and medial (M) directions. In the inset is an image of latex-filled brain vasculature taken from Scremin [1], with a box that indicates the approximate size and location of a typical craniotomy and an arrow that identifies the MCA. (B) Tracing of the surface arterial vascular network from the image in (A). Branches of the MCA are indicated, as are representative examples of the communicating arterioles (CA) that form the surface network and diving arterioles (DA) that supply cortex. (C) Maximal projection of a TPLSM image stack through a cortical arteriole. The dark line indicates the location where the line-scan data were taken, and the arrow represents the direction of flow obtained from these scans. (D) Line-scan data from the vessel in (C) to quantify the flow of RBCs. Each scan is displayed below the previous one, forming a space–time image with time increasing from top to bottom of the image. The dark streaks running from upper right to lower left are formed by the motion of the non-fluorescent RBCs. The RBC speed is given by the inverse of the slope of these streaks; the direction of flow is discerned from the sign of the slope. (E) RBC speed along the center of the arteriole shown in (C) and (D) as a function of time. The periodic modulation of the RBC speed occurs at the approximately 6-Hz heart rate. The dotted line represents the temporal average of the speed. (F) RBC speed in an arteriole, averaged over 40 s, as a function of the transverse position in the vessel along horizontal *(y)* and vertical *(z)* directions. The parabolic curve represents the laminar flow profile that most closely matches the data, i.e., *s* = *A*·(1 − *r*/*R*)^2^ where *s* is the speed of the RBCs, r is a radius from the origin and corresponds to either the *y* or *z* direction, R is the measured vessel radius of 26 μm, and *A* is a free parameter (A = 10 mm/s).

Large-scale maps of the fluorescent brain vasculature in the field of a craniotomy (Materials and Methods) (Figure S1) reveal distal branches of the MCA as well as communicating and diving arterioles (Figure 1A). A high degree of redundancy in the surface arteriole network is apparent, with anastomoses formed between vessels from both the same and different MCA branches (Figure 1B). Candidate vessels for photothrombotic clotting are identified from the maps (Figure 1A) and traced back to a readily identifiable artery or vein; we considered only target vessels that are arterioles. High-resolution planar images (Figure 1C) are used to determine the baseline diameter of the target as well as of the vessels that lie upstream and downstream from the target. Line scans, in which the laser focus is repetitively scanned along the axis of each vessel (Figure 1C), are used to form a space–time image in which the non-fluorescent RBCs are represented as dark streaks (Figure 1D) [14,15,23,24]. The sign and magnitude of the slope of the streaks reflect the direction and speed, respectively, of RBC motion (Figure 1D, Figure S2, and Protocol S1). The analysis of this data yields a time series of the RBC speed in an arteriole (Figure 1E). We average over time, typically 40-s epochs, to suppress the underlying cardiac contributions (Figure 1E). We observe that the average speed increases with increasing vessel diameter (Figure S3), consistent with previous studies [25].

The line-scan method is accurate for large vessels only if the speed varies slowly across the diameter of the vessel. We thus measured the spatial profile of the average speed across both the lateral *(y)* and axial *(z)* directions and observe a profile that peaks in the middle of the vessel and smoothly decreases toward the vessel wall (Figure 1F). The observed profile is flatter than that for laminar flow of a Newtonian fluid in a stiff pipe (Figure 1F), as expected for particle flow in a soft vessel [26]. The smooth nature of the spatial variation in speed implies that the extraction of speed from the line-scan measurements is relatively insensitive to misalignment between the scan and vessel axes so long as the measurements are performed close to the center of the vessel.

**Figure 2:**
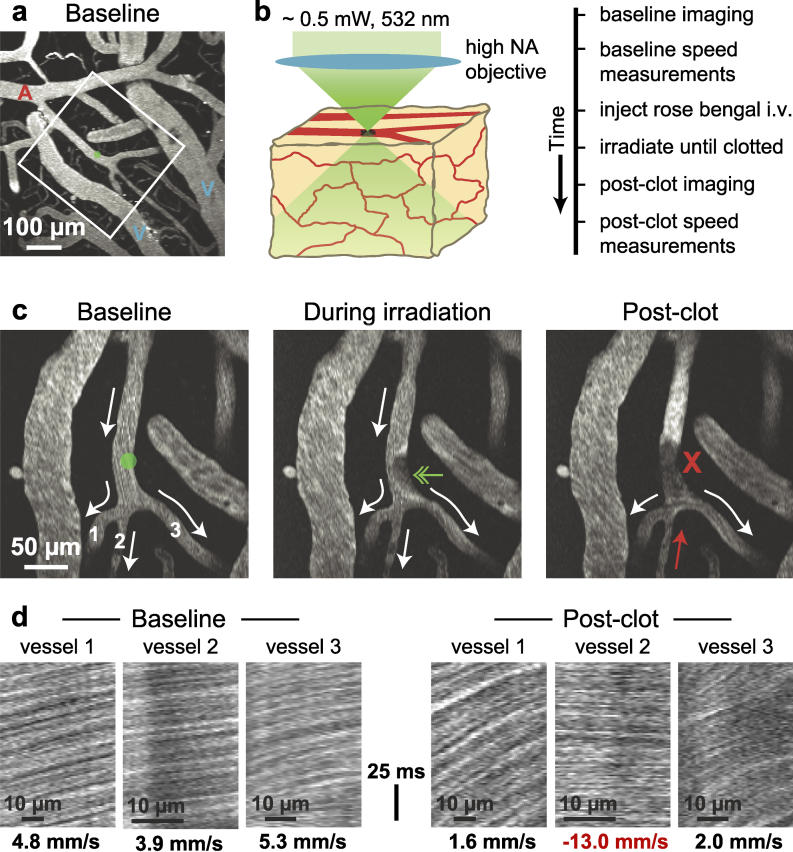
Photothrombotic Clotting of Individual Targeted Surface Cortical Blood Vessels in Anesthetized Rat (A) Maximal projection of TPLSM image stack showing several surface arterioles (red A) and venules (blue V). The green circle indicates the region of the targeted arteriole that will be irradiated with green laser light. The white box indicates the region and orientation of the images in (C). (B) Schematic illustration of the targeted photothrombotic occlusion of a vessel and experiment timeline. After baseline imaging and blood flow measurements, rose bengal is intravenously injected into the animal. Green laser light is focused onto the wall of the target vessel, which excites the rose bengal and ultimately triggers the natural clotting cascade. Surface vessels adjacent to the target vessel are not occluded because they are not exposed to the 532-nm irradiation. (C) Planar TPLSM images of photothrombotic clotting of a surface arteriole. The frame on the left is taken at baseline. The green circle indicates the region of the targeted arteriole that will be irradiated, whereas the white arrows indicate the blood flow direction, as determined from line-scan measurements in the targeted vessel and in the vessels downstream from the target. The numbers over the downstream vessels correspond to the numbered line-scan data shown in (D). The streaked appearance of the vessels is due to the motion of RBCs during the acquisition of the image. The center frame is taken after an intravenous injection of rose bengal and 2-min irradiation with 0.5 mW of 532-nm laser light. The vessel is partially occluded (indicated by green double arrow). The right frame is taken after one more minute of irradiation. The target vessel is completely clotted (indicated by red X) whereas surrounding vessels are unaffected. Stalled blood flow is indicated by the dark mass of clotted cells in the target region and the brightly fluorescent region of blood plasma upstream from the target region. Note that blood flow is maintained in the branches downstream from the target vessel by a reversal in the direction of blood flow in the center branch, as determined from the line-scan data in (D). (D) Baseline and post-clot line-scan data for the numbered vessels downstream from the target vessel shown in (C). The average RBC speed determined from the line-scan data is indicated for each case, with a positive speed taken to be along the baseline direction of flow.

Once the baseline measurements of vessel diameter and RBC speed were completed, the photosensitizer rose bengal was added to the blood stream (Methods). Green laser light, tightly focused on the target vessel (Figure 2A), was used to excite the dye (Figure 2B). We monitored the clot formation in real time, as illustrated for the example of flow into a trifurcating vessel in Figure 2 (Figure 2C; Video S1), and adjusted the laser power to near-threshold levels for clot formation. Once the clot was completed, the diameters of neighboring vessels and the speed of flow in these vessels was again measured (Figure 2D). Further, in control experiments the RBC speed decreased only slightly (5% per h) over 3 h (Figure S4), which far exceeds the duration of a set of measurements after a clot.

### Localization of Photothrombotic Effects

A combination of in vivo flow measurements and post-mortem histology was used to determine the effects of focal irradiation of the target surface vessel on non-targeted surface vessels and the underlying cortical parenchyma. Surface vessels located lateral to the target vessel were never observed to clot, consistent with their exposure to insufficient laser power to trigger photothrombosis (Figure 2C). To reduce parenchymal damage deep to the irradiation site, the incident laser power on subsurface vessels was minimized by three physical mechanisms: (1) the absorption of incident light by the target vessel; (2) the divergence of the beam beyond the target vessel as a result of the strong focus; and (3) the scattering of light by brain tissue. Flow measurements were assessed in parenchymal capillaries situated less than 150 μm beneath the target vessel and within a lateral distance of 100 μm from the target vessel (55 capillaries, across six clots in four rats). In addition, flow was measured in control capillaries located at the same depth but 2 mm or more from the target vessel (18 capillaries). Flow was measured at baseline and after occlusion of the target vessel. The average flow speed across all capillaries was reduced to 57% ± 8% (mean ± standard error of the mean) of the baseline value and was highly variable (standard deviation = 64%). This reduction appeared to result from an increase in the normal occurrence of capillary stalls [14,27], from a baseline level of 8% to a post-clot level of 30%. Flow in control vessels was 84% ± 5% of the baseline value, consistent with slow rundown over the course of the experiment (Figure S4), and was less variable (standard deviation = 20%). In toto, these control data show that neighboring surface arterioles and venules remain unclotted after a target vessel is occluded. Further, clots in sub-surface capillaries are limited to vessels that lie directly beneath the target vessel, typically far from the downstream region perfused by the surface vessel.

**Figure 3:**
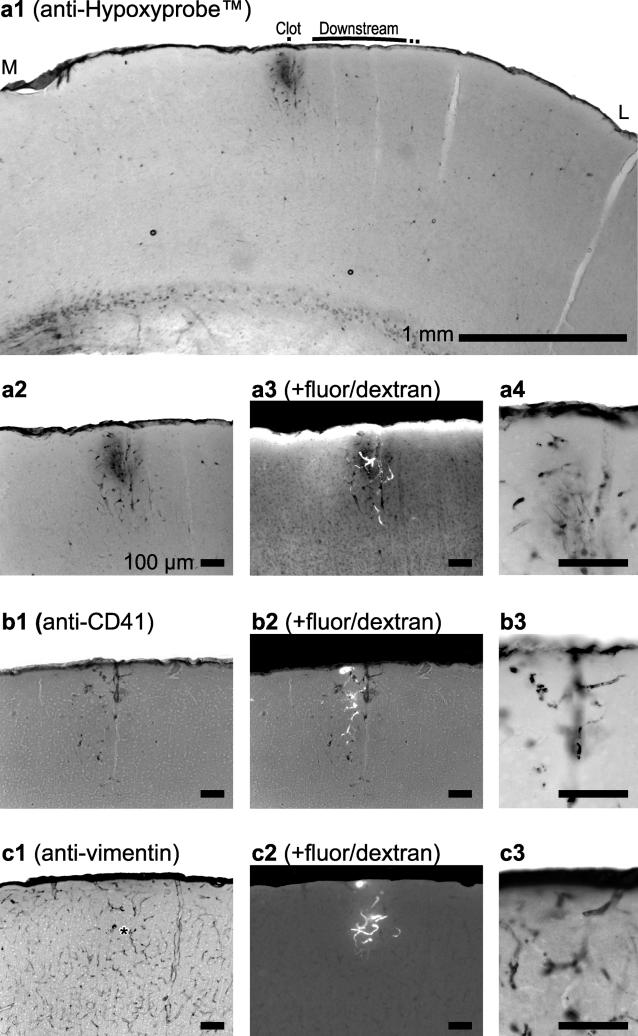
Assay of Oxidative Stress and Vascular Disruption Pimonidazole hydrochloride was introduced into the bloodstream at 1 h after targeted photothrombosis. At the end of this period, the animal was euthanized and transcardially perfused for brain tissue fixation. Labeling is illustrated for a closely spaced series of sections (within 150 μm). (A1–A4) The section, immunolabeled with an antibody against pimonidazole adducts (Hypoxyprobe), shows localization of adducts at uptake sites that are largely restricted to zones beneath the clot. Lateral and medial directions are labeled by M and L, respectively. The intermediate- and high-magnification views show immunolabeling across neural compartments, including parenchyma and blood vessels (A2 and A4). A mixed brightfield-fluorescent image shows that the fluorescein-dextran retained in the vessels and extravasated into the parenchyma overlaps the pimonidazole labeling (A3). (B1–B3) Immunolabeling of the platelet marker CD41 indicates sparse damage. A mixed image of fluorescein-dextran vascular retention and the CD41 immunoreactivity indicates that the damage is confined to just below the clot (B2). (C1–C3) Immunolabeling of tissue with the reactive astrocyte marker vimentin increased only marginally the labeling of vessels just below the clot (indicated by an asterisk). A mixed image of fluorescein-dextran retention and the vimentin immunoreactivity indicates that the damage is co-localized (C2).

We assessed acute ischemic effects of photothrombosis across cell types with the hypoxia marker pimonidazole hydrochloride (Hypoxyprobe; Chemicon International, Temecula, California, United States). This probe forms an immobile adduct in viable but hypoxic tissue, that is visualized post hoc by antibody staining [28]. This marker was injected intravenously after clot formation and allowed to circulate for 1 h prior to sacrifice. As illustrated by the example of Figure 3A, the cortical distribution of Hypoxyprobe is highly sequestered in vessels and cells immediately below the photothrombotic clot (Figure 3A2 and 3A4) within a volume of ~500 pl (four clots across four animals). Additional analysis revealed an increased tendency for the retention of fluorescein-dextran in the extracellular space that borders vessels that lie directly beneath a photothrombotic clot (Figure 3A3). In control experiments, we observed that irradiation of the brain surface in a region between surface vessels also led to fluorescein-dextran retention in subsurface capillaries (Figure S5). These data imply that the pathology seen in capillaries beneath the target vessel is the result of photochemical damage, consistent with previous studies [29] and with our findings of increased capillary stalls beneath the target vessel. Critically, the territory of labeled Hypoxyprobe and fluorescein retention overlap (Figure 3A3). Thus there was no indication of wide-spread tissue hypoxia as a result of the focal clot formation in the surface arteriole.

To probe ischemic cascades in hypoxic parenchymal vessels, we localized activated platelets by immunostaining with CD41 antibodies. Anti-CD41 immunoreactivity is also seen just below the clot (Figure 3B1 and 3B3) and overlaps with retention of fluorescein-dextran (Figure 3B2). To assay potential damage to other neural elements, we probed for reactive astrocytes by immunostaining for the filamentous astrocyte protein vimentin, as this is strongly up-regulated in reactive astrocytes that proliferate in response to injury [30]. Vimentin immunostaining is relatively evenly distributed in the cortex, most likely in glial endfeet on vessels, but shows only negligibly increased staining (Figure 3C1 and 3C3) among those ischemic vessels that retain fluorescein (Figure 3C2). Together these data indicate that photothrombosis results in a restricted zones of marked vascular ischemia (five clots across five animals).

**Figure 4:**
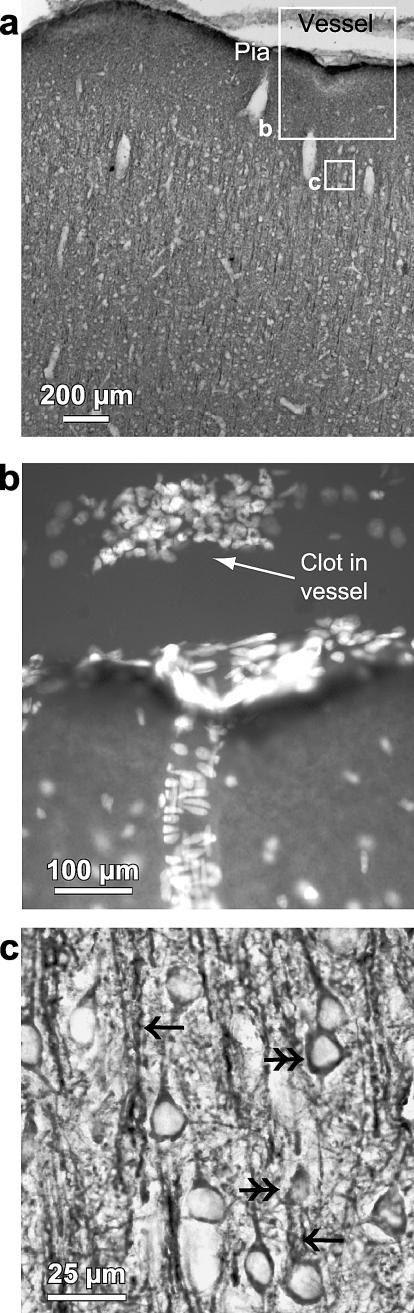
Neuronal Integrity near Photothrombotic Clot The animal was sacrificed 1 h after the disruption, and the tissue was harvested from below the target vessel shown in Figure 5C. (A) The section, immunolabeled with an antibody against MAP2, shows widespread staining of neurons. (B) Fluorescent imaging of propidium iodide counterstain. The stained endothelial cells demarcate vessels. Note the numerous nuclei with segmented lobes characteristic of aggregates of leucocytes within the vessel (clot). (C) High-magnification view shows subtle neuropathology in some cells, i.e., corkscrew dendrites (single arrows) and shrunken neurons with eccentric nuclei (double arrow).

To selectively assess neuronal integrity in the vicinity of photothrombosis, we stained tissue sections with antibodies that recognize microtubule associated protein 2 (MAP2); MAP2 staining intensity is a sensitive and early indicator of neuropathology associated with ischemia [31]. As illustrated by the example in Figure 4, relevant tissue could be localized near a clotted vessel that remained intact (Figure 4A) and contained intra-luminal aggregates of nucleated blood cells (Figure 4B). The tissue near the clotted vessel exhibits near uniform staining for MAP2 (Figure 4A). Only subtle neuropathology is observed below the clot as indicated by a slight loss of staining for MAP2 in layer 1 (Figure 4A) and angulated neuronal somata with eccentric nucleus location and a corkscrew appearance in scattered dendrites (Figure 4C). In general, there was no indication of severe neuropathology in neurons that lay directly beneath the targeted vessel (seven clots across three rats) (Figure S6). Thus, we have achieved the desired goal of a localized clot to a single surface vessel with minimum collateral damage.

### Qualitative Aspects of Flow Rearrangement after a Single Arteriole Occlusion

The essential consequence of an occlusion to a surface arteriole is illustrated by the example in Figure 2. We observe that blood flow downstream from the occlusion is maintained through a reversal in the direction of flow in a vessel at the first downstream branch (middle branch, Figure 2C and 2D). The reversal occurs within 1 s, i.e., within the acquisition time of an image, after the target vessel was occluded. As a population across all experiments (47 clots in 34 rats with arterioles that ranged from 15 to 140 μm in diameter), flow reversal occurred in one vessel at the first downstream branch after 100% of the clots. In a subset of targeted vessels (*n* = 7), the clot broke free midway through the experiment and had to be reformed. In each of these cases, the pattern of downstream flow observed after reclotting was the same as that observed after the first clot.

**Figure 5:**
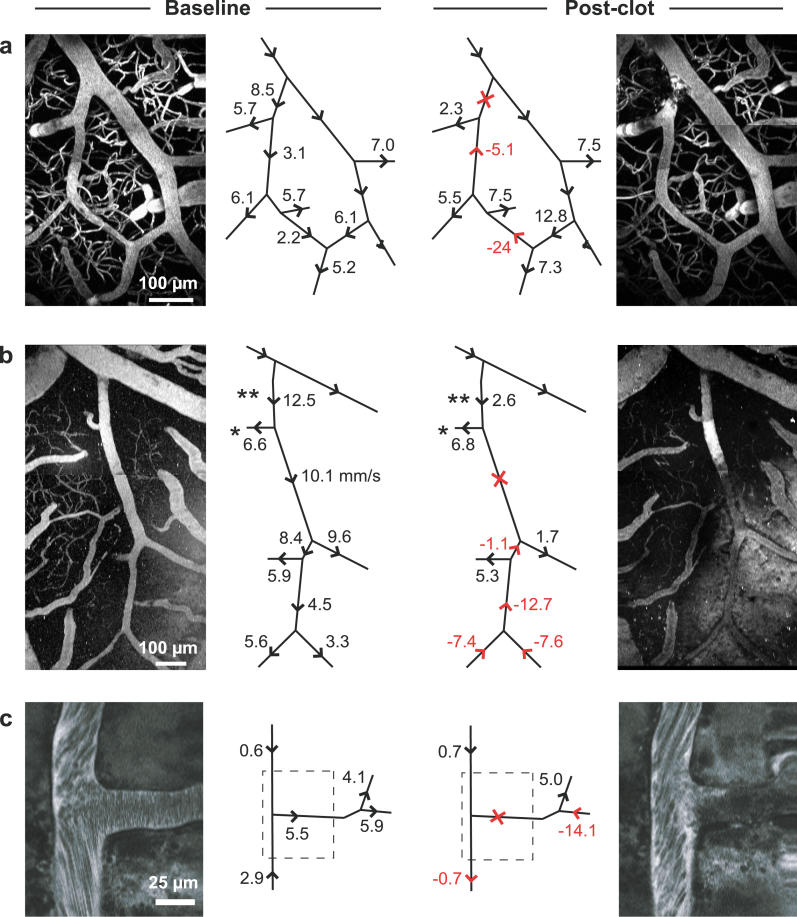
Examples of Flow Changes that Result from Localized Occlusion of a Cortical Surface Arteriole (A–C) On the left and right are TPLSM images taken at baseline and after photothrombotic clotting of an individual vessel, respectively. Left center and right center are diagrams of the surface vasculature with RBC speeds (in mm/s) and directions indicated. The red X indicates the location of the clot, and vessels whose flow direction has reversed are indicated with red arrows and labels. In the examples of panels (A) and (B) we show maximal projections of image stacks whereas the example in panel (C) shows single TPLSM planar images; the streaks evident in the vessels in these latter frames are due to RBC motion, and the dashed box in the diagrams represents the area shown in the images.

Three additional examples illustrate typical flow changes in vessels upstream as well as downstream from an occlusion of a communicating arteriole (Figure 5; RBC speeds are shown for baseline and post-clotting conditions). In the circular architecture of Figure 5A, the reversal of flow direction at the first branch downstream from the clot is accompanied by a decrease in the speed of the RBCs. An anastomosis that connects to the same MCA branch as the targeted vessel is clearly the origin of this reversed flow. In the tree-like structure of Figure 5B, the reversal of flow direction at the first branch downstream is again accompanied by a decrease in RBC speed. A complicated pattern of reversed and non-reversed flow is observed in vessels farther downstream from the clot. In addition, flow is slowed in the upstream vessel that originally was a source to the targeted vessel (** in Figure 5B), whereas flow is essentially unchanged in a parallel vessel that shares the same source as the targeted vessel (* in Figure 5B). For the final example (Figure 5C), the targeted vessel was originally fed by the confluence of flow from two branches of the MCA. A photothrombotic clot results in a reversal in downstream flow, as well as a reversal in flow in one of the vessels that was a source of blood to the target vessel. The confluence of separate sources was observed to move to another point along the anastomosis between the MCA branches.

### Quantitative Aspects of Flow Rearrangement after a Single Arteriole Occlusion

**Figure 6:**
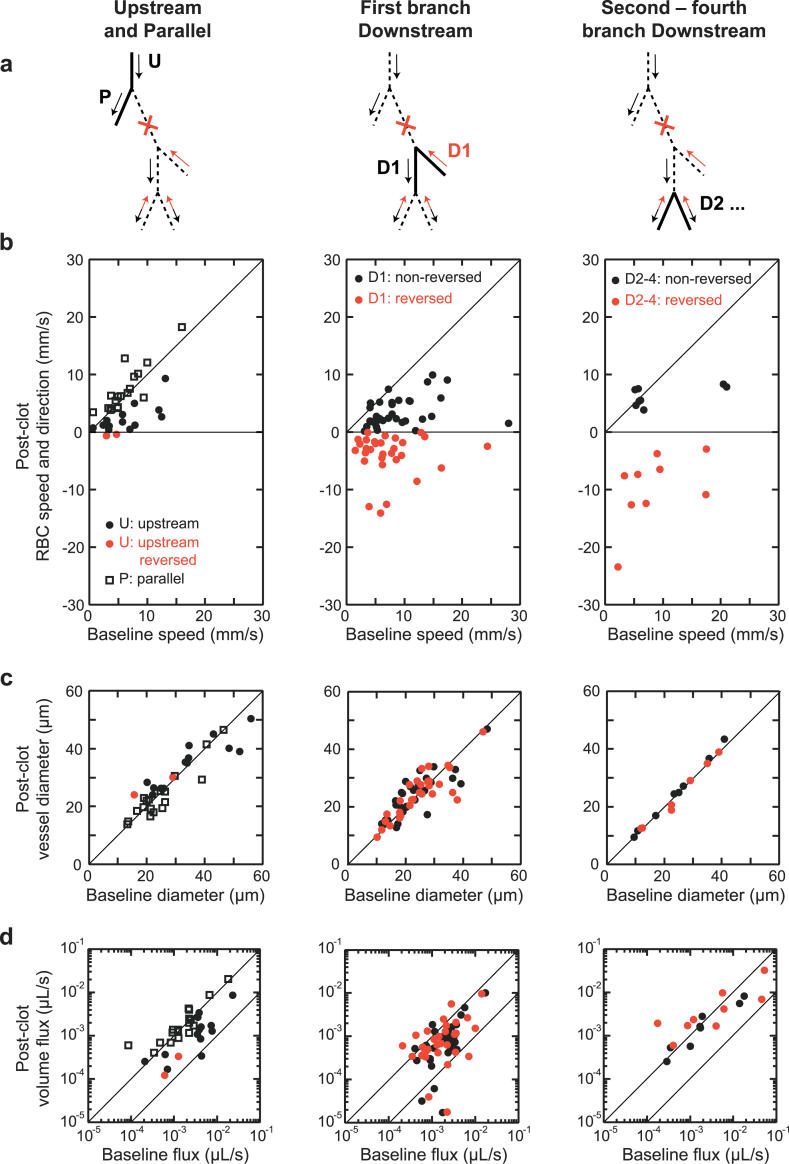
Compendium of Flow Changes following Localized Photothrombotic Clotting of Communicating Surface Arterioles (A) Illustration of the four different classes of vessels considered, each delineated by their connectivity to the target vessel (indicated by an X). (B) Plots of post-clot RBC speed as a function of baseline RBC speed for each vessel class. The post-clot and baseline speeds were significantly correlated for the upstream and parallel vessels, but uncorrelated for the downstream vessels (Table 1). (C) Plots of post-clot vessel diameter as a function of baseline diameter. The pre- and post-clot diameters were correlated for all cases (Table 1). (D) Plots of post-clot volume blood flux as a function of the baseline value. The diagonal lines represent post-clot flux levels of 10% and 100% of baseline. As for the diameters, the pre- and post-clot fluxes were correlated for all cases (Table 1).

We now consider the systematics of changes in RBC speed as well as potential changes in vessel diameter that resulted from occlusion of the target. Vessels in the neighborhood of the target vessel were classified according to their topological relationship to the target (Figure 6A): Upstream vessels (U) provided a source of blood to the target vessel; parallel vessels (P) shared the same source as the target; downstream vessels drained from the target vessel and are grouped as those immediately downstream (D1) and those two to four branches downstream (D2 to D4) from the target. We found that the RBC speed in upstream vessels slows substantially, yet the RBC speed in parallel vessels was increased slightly (Figure 6B, left). In two cases the target vessel was fed by a confluence of sources, and one of the upstream vessels reversed direction (as in Figure 5C). For vessels that lie at the first downstream branch from the target, the average RBC speed is reduced and half of the vessels reverse their flow direction (Figure 6B, middle). For vessels that lie farther downstream, the average speed is unchanged and the flow direction is reversed in approximately half the vessels (Figure 6B, right). Summary statistics are given in Table 1.

**Table 1:**
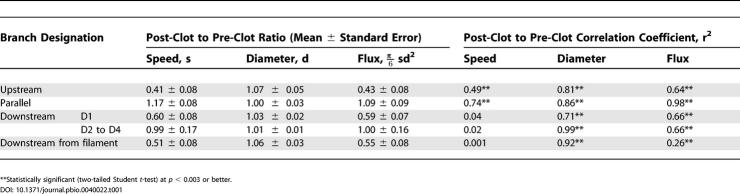
Summary Statistics for Changes in Vascular Parameters

In contrast to the substantial changes in the speed of RBC flow in vessels in the neighborhood of the target vessel, only small changes are induced in the diameter of individual vessels as a result of clotting the target vessel (Figure 6C). On average, the diameter of all classes of vessels remained unchanged by an occlusion (see Table 1).

Our measurements of RBC speed, denoted *s,* and vessel diameter, denoted *d,* may be combined to calculate the volume flux of blood, *F,* where 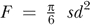 under the approximation of laminar flow. We find that the flux is reduced by ~55%, relative to the baseline value, for upstream vessels and slightly increased for parallel vessels (Figure 6D, left, and Table 1). For vessels downstream from the clot, the post-clot flux ranges between 5% and 200% of the baseline value, with the flux reduced by approximately 40% at the first downstream branch (Figure 6D, center, and Table 1) and unchanged for vessels lying farther downstream (Figure 6D, right and Table 1). We estimate that random errors in the measurement of speed and diameter contribute a 10% uncertainty to the estimate of the flux for each vessel and that drift in the flux contributes an additional 10% uncertainty (see Figure S4 and Protocol S1). The observed spread in the baseline and post-clot values of the flux (Figure 6D and Table 1) is thus dominated by biological variability. Lastly, the changes in flux were correlated with changes in RBC speed for all vessels (*p* < 0.001), but the changes in flux were correlated with changes in vessel diameter only for the case of parallel vessels (*p* < 0.01).

### Flow Rearrangement after Occlusion of the MCA

**Figure 7:**
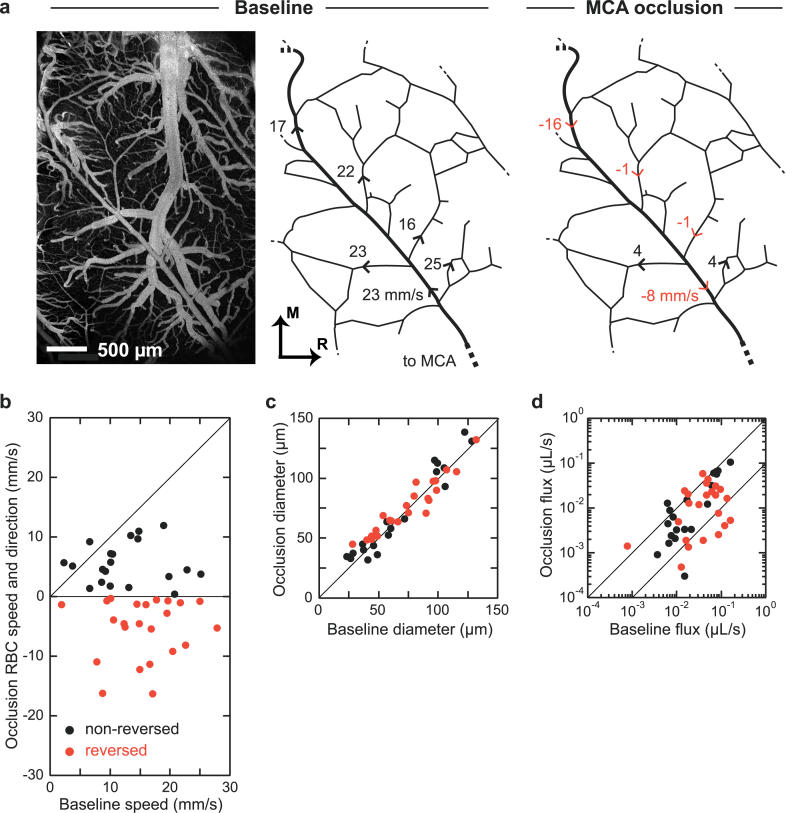
Quantitative Measurements of Flow Changes in Cortical Arterioles after Filament Occlusion of the MCA (A) Example of flow changes observed following MCA occlusion. Left: projection of a TPLSM image stack taken at baseline. Center: tracing of the surface arteriole network from the image with the baseline blood flow speed and direction indicated in some vessels. Right: blood flow speed and direction during MCA occlusion. Red arrows and speed labels indicate vessels whose direction has reversed. The axes indicate the rostral (R) and medial (M) directions. (B–D) RBC speed, vessel diameter, and volume blood flux, respectively, during MCA occlusion as a function of baseline values. The baseline and occlusion values of the diameter and flux are significantly correlated (*p* < 0.005), with r^2^ = 0.92 and 0.26, respectively; the baseline and occlusion values of the speed are not significantly correlated.

As a means to probe the possible ubiquity of reversals in flow in the arteriole communicating network, we studied the effects of a filament occlusion of the MCA on the flow in individual surface arterioles (Figure 7A). This artery is the primary supply to the surface network. The arterioles measured in response to MCA occlusion were, on average, of larger diameter than those measured in response to photothrombotic occlusion. Similar to the case of the localized occlusion (see Figure 2), we measured the speed of RBC flow and the diameter of vessels before and after occlusion of the MCA. The example data of Figure 7A, in which flow velocities are indicated in six of the surface arterioles, illustrate the essential results. The speed of RBCs was substantially reduced in five of these arterioles, consistent with a drop in the source perfusion. Critically, the direction of flow in four of the six arterioles was reversed after the occlusion.

As a population across all measurements (42 vessels across 11 rats), we observed an overall reduction in the magnitude of RBC speed and the presence of vessels with reversed as well as non-reversed flow in each experiment (Figure 7B). The diameter of the vessels was essentially unaffected by the occlusion (Figure 7C and Table 1), and there was no correlation between changes in the volume flux and vessel diameter for individual vessels. The volume flux was reduced by 45% after the occlusion (Figure 7D and Table 1). On average, approximately half the vessels reversed flow direction during the MCA occlusion.

## Discussion

**Figure 8:**
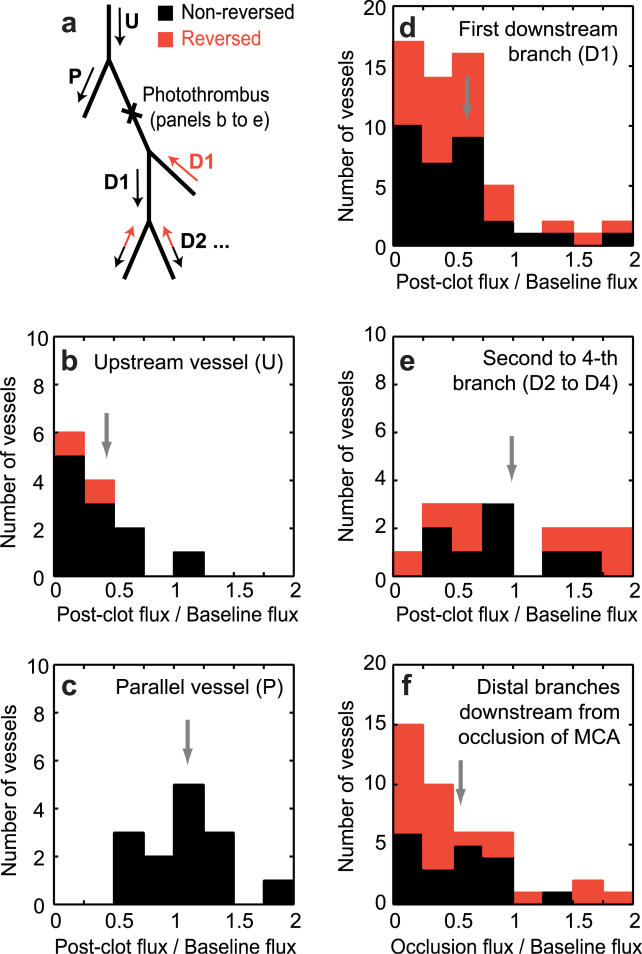
Summary of Quantitative Measurements of Changes in Volume Blood Flux in Response to Single Microvessel Occlusion and MCA Occlusion (A) Illustration showing topological relationship of vessels in (B) to (E) relative to the clotted arteriole (indicated with an X). (B–E) Histograms of the ratio of the post-clot flux to the baseline flux for vessels with different topological relationships to a photothrombotically clotted cortical surface arteriole: (B) upstream, (C) parallel, (D) first branch downstream, and (E) second to fourth branch downstream. (F) Histogram of the ratio of the flux measured during intra-luminal filament occlusion of the MCA to the baseline flux for cortical arterioles. In the interests of clarity, outliers with post-clot flux greater than twice the baseline flux were excluded from these histograms: one parallel vessel (ratio = 7), two D1 vessels (ratio = 2.5, 2.8), one D2 – 4 vessel (ratio = 11), and one filament occlusion vessel (ratio = 2.1). The arrows point to the mean values across all data points for each vessel class.

We examined the resilience of blood flow in a network of communicating arterioles that lies in the territory fed primarily by branches of the MCA (see Figure 1). Blood flow downstream from a targeted, localized occlusion does not stop, but rather is reestablished at the first downstream branch by a reversal in the direction of flow in one of the downstream branches (see Figures 2 and 5). Such flow reversals are common phenomena downstream from localized microvessel occlusions (see Figure 5). They are also a feature within this territory in response to occlusion of the MCA (Figure 7). The magnitude of the blood flow change following a localized occlusion in a surface arteriole depends on the topological relationship of a vessel to the clotted vessel (see Figure 6). The average flow at the upstream and the first downstream branch is reduced by less than half, whereas the average flow in vessels parallel to the clotted vessel and far downstream from the occlusion remain near baseline levels (Figure 6). In general terms, this study has revealed the persistent nature of perfusion at the level of cortical surface communicating arteriole networks (Figure 8).

Our observation that, on average, the diameters of vessels remained constant after localized microvessel occlusion (see Figure 6C and Table 1) is somewhat surprising. Complementary studies on the acute effects of mechanical occlusion on vessel diameter report that the cessation of flow in MCA tributaries, realized through multiple ligations to the surface branches [32], leads to an immediate, apparoximately 20% increase in vessel size. An even larger increase has been reported immediately following the occlusion of the common carotid arteries [33]. In contrast, a decrement in diameter of the MCA is seen after its occlusion by photothrombotic formation of a large, approximately 1.5 mm–long clot [34]. On the chronic timescale of 1 mo, multiple ligations to the surface branches of the MCA led to dilation to 200% of the initial vessel diameter [35]. In addition, other work has shown that cerebral arterioles, especially leptomeningeal anastomoses that connect the anterior cerebral with the MCA [5], show an approximately 50% increase in diameter 1 mo after MCA occlusion [36,37]. The dilation observed in previous acute and chronic studies has been attributed to active vascular regulation and/or to remodeling mechanisms. In contrast to these past results, we observe no systematic change in the diameter of downstream pial arterioles over a few hours in response to a small, approximately 100 μm–long clot formed in single surface arterioles by photothrombotic occlusion (Figure 6C and Table 1). One possible mechanism for this observed stability in vessel diameter may be that the new flow pattern is rapidly established, in about 1 s after the formation of an occluding clot (Video S1). Of interest, a classic means to demonstrate vascular auto-regulation in humans is based on modulation of the partial pressure of inspired CO_2_. However, recent work now shows that although cerebral blood flow undergoes predictable changes, the diameter of large cerebral arteries may be unchanged [38,39] or vary widely [40].

With regard to the magnitude of reperfusion in the vicinity of a localized clot, the observed flux was 60% of normal flow at the first downstream branch and 100% of normal flow at more distal downstream branches (Figures 6 and 8). The results of past studies suggest that flow rates must drop to 10% to 30% of baseline values [41–43] before irreversible neuropathology occurs. Such damage is neither expected nor observed to occur under the conditions of this study (see Figures 3 and 4), consistent with a past study of distal MCA occlusions [44]. In particular, we observed limited pathology directly below the clotted surface vessel, which was caused by photochemical damage to the capillaries, and no pathology in cortical regions that lie downstream from the site of clot formation (Figures 3 and 4). Thus the surface network of communicating arterioles appears to be well protected against single-point occlusions of surface arterioles by virtue of its architecture alone.

The redundancy of brain vasculature changes as one proceeds from the level of the internal carotid arteries to that of the brain capillaries. At the level of the supply to the brain, the Circle of Willis provides sufficient redundancy so that all cephalic arteries are perfused in the event of an occlusion to one of the internal carotids. Blockages immediately downstream from the Circle of Willis, at the level of a cerebral artery, lead to a variety of neuropathologies. In particular, an occlusion to the base of the MCA typically results in infarction and widespread cell death in the basal ganglia whereas the same blockage produces less-severe deficits in the cerebral cortex or penumbral region [42,44]. Compensation at the level of cortex has been attributed to flow through leptomeningeal anastomoses that connect the territories of the intact anterior cerebral artery and the MCA [5]. The present work demonstrates that functional compensations can also occur at the scale of surface communicating arterioles, formed through anastomoses between branches of the MCA (Figure 1A and 1B), since perfusion is maintained in neighboring branches after photothrombosis of a single arteriole (see Figures 5, 6, and 8). This failsafe mechanism most likely results from the many short-range loops in this arterial network, which may also play a role in rerouting a fixed supply of blood among different cortical columns during shifts in cortical electrical activity [45]. The highly redundant cortical surface vasculature discussed here for rats is also present in humans [4,46]. In contrast to the present results for the surface vasculature of cortex, the apparent lack of a similar system of anastomoses in the basal ganglia may contribute to its greater vulnerability after a blockage of the MCA [47].

The present work focused on the surface network of communicating arterioles (Figure 1B, CA), a two-dimensional network formed by anastomoses between branches of a cerebral artery. This network delivers blood to the cortical parenchyma through a series of diving arterioles (Figure 1B, DA). The topology and resilience of the three-dimensional, subcortical microvascular network remain an open issue. From a technical perspective, the present methodology to form localized occlusions is ideal for surface vessels but cannot selectively target vessels at depth because, as for all techniques that rely on the absorption of a single photon, all vessels superficial to the target will also be affected. Thus a technology that depends on the nonlinear absorption of light is required. Such a technique, which makes use of high-fluence ultrashort pulses of light that can precisely ablate tissue [48], has been demonstrated to work with vessels in superficial cortical layers [49]. This method cannot, in turn, be used to target clots in the surface communicating arteriole network, as the lack of containment by parenchymal tissue causes irradiated vessels to burst. Preliminary data suggest that clots to subsurface microvessels leads to perfusion failure in the immediate downstream vessels. This is in marked contrast to the redistribution of flow that preserves perfusion in all downstream branches of the surface communicating arteriole network (see Figures 6, 8D, and 8E).

In humans, damage to microvessels is a known pathological condition [50–54]. In particular, occlusion of small-scale arterioles is a likely cause of clinically silent lacunar infarcts [55] that are correlated with an increased risk of dementia and cognitive decline [56–58]. It is thus interesting that the Rotterdam Scan study [57], which identified clinically silent lacunar infarcts through magnetic resonant imaging, found that few cortical infarcts were located near the surface and thus where the vasculature appears to be most redundant [46]. Our results for surface blood flow dynamics (see Figures 6, 7, and 8) suggest an emerging relation between vascular topology and susceptibility to stroke in different regions of the brain.

## Materials and Methods

### Surgery

Our subjects were 51 Sprague-Dawley rats. In 34 animals of both sexes, 150 to 350 g in mass, one to five individual microvessels were occluded photothrombotically. In 11 male animals, 290 to 310 g in mass, the MCA was occluded by the filament method. The remaining six rats were used for control experiments. All animals were anesthetized by interperitoneal injection of urethane (150 mg per 100-g rat), supplemented as necessary; urethane is reported to maintain autoregulation of cerebral blood flow [59]. Atropine sulfate was delivered by subcutaneous injection (5 μg per 100-g rat) at the start of surgery and supplemented hourly at a reduced dose (1 μg per 100-g rat) to reduce secretions. We further supplemented by subcutaneous injection of 5% (w/v) glucose in phosphate buffered saline (PBS) (0.5 ml per 100-g rat) every hour. Body temperature was maintained at 37.5 °C and heart rate was continuously monitored. A 4-mm by 6-mm craniotomy was prepared over parietal cortex, with the center at medial-lateral equal to 4.5 mm and anterior-posterior equal to −4.5 mm relative to Bregma. The dura was removed, a chamber consisting of a metal frame and a removable coverglass (no. 1) lid was glued to the skull [60], and the space between the exposed brain surface and the coverglass was filled with 1.5% (w/v) low–melting point agarose in an artificial cerebro-spinal fluid [61]. A 0.3-ml bolus of a 5% (w/v) solution of 2 MDa fluorescein-conjugated dextran in PBS was injected into the tail vein to label the blood plasma. The care and experimental manipulation of our animals have been reviewed and approved by the Institutional Animal Care and Use Committee at the University of California, San Diego.

### TPLSM

Images were obtained with a two-photon laser scanning microscope of local design [62] that was modified to include a path for a green laser beam (see Figure S1 and Protocol S1). A 0.12 numerical aperture (NA), 5×-magnification air objective was used to obtain images of the surface vasculature across the entire cranial window to aid in navigating around the cortical vasculature. We changed to a 0.8-NA, 40×-magnification water-immersion objective for high-resolution imaging, line-scan measurements, and photothrombotic clotting with the green laser. The line-scan rate was 1.3 kHz for measuring RBC speed, and we typically acquired line-scans for 40 s and report the average speed over this period.

### Photothrombotic clotting

A continuous wave green-light laser (λ = 532 nm) (TIM-622; Transverse Industries, Taipei Hsien, Taiwan) was directed onto the beam axis of the microscope with a dichroic mirror (see Figure S1). The green-light beam underfilled the back aperture of the objective and was aligned so it focused at the center of the same plane as the near-infrared pulsed laser beam and formed an approximately 5-μm diameter spot. As the green-light overlapped with the fluorescent spectrum of fluorescein, the green-light was delivered in pulses of 1-s duration that were interspersed with TPLSM imaging.

Vessels targeted for clotting were centered in the imaging field (see Figure 2A). The rat was then given a 0.3-ml intravenous injection of 1% (w/v) rose bengal (Na salt) in PBS, and the wall of the target vessel was irradiated with 0.1 to 5 mW of green laser light for a total of 30 to 600 s. Irradiation of a photosensitizer leads to the production of singlet oxygen [63], which damages the wall of the vessel and subsequently triggers a clotting cascade that leads to an occlusion [16,17]. We slowly increased the green laser power while monitoring clot formation in near real time and used the minimum power required to trigger clot formation in the target vessel. This procedure led to the formation of a single, localized clot (see Figure 2C and Video S1). In control experiments, green laser irradiation at a typical power led to no visible effects on the target vessel in the absence of rose bengal (Figure S7) and no observable retention of fluorescein-dextran in the tissue below the target vessel (see Figure S5).

### Filament occlusion of MCA

A filament is advanced inside the internal carotid artery until it blocks the origin of the MCA, as described [21]. Briefly, an incision is made in the neck, exposing the left common carotid artery. The external carotid and pterygopalatine arteries are ligated with 4–0 silk suture. An incision is made in the wall of the common carotid artery and a 4–0 nylon suture, whose tip has been blunted by heating on a microforge, is advanced 17.5 mm from the bifurcation point of the external and internal carotid arteries, thereby blocking the ostium of the MCA. Flow velocities are measured at baseline and during the MCA occlusion. A large-scale TPLSM image (see Figure 7A) is used to ensure the same vessels are measured at baseline and during the occlusion. The occlusion measurements are performed within two hours of the insertion of the filament.

### Postmortem histology

The cortex of seven of the animals that received localized photothrombotic clots, four with pimonidazole hydrochloride (Hypoxyprobe-1 [90201]; Chemicon) injected 1 h before sacrifice, were perfused transcardially with 100 ml of PBS, followed by 100 ml of 4% (w/v) paraformaldehyde in PBS. Fiducial marks were made in the corners of the craniotomy by passing −20 μA though a single tungsten electrode that translated at 2 mm/s. The brain was cryoprotected with sucrose and then 50 μm–thick sections were cut in a coronal plane on a freezing-sliding microtome.

Sections near the location of the clots were selected based on the location of the targeted vessels relative to the fiducial marks, as determined from widefield images of the craniotomy taken before and after the perfusion and the retention of fluorescein-dextran in capillaries beneath the target vessel (see Figure 3A3). To confirm the completeness of a clot in the surface vessels, we looked for intra-luminal aggregates of blood-borne white cells by staining sections with propidium iodide (1 μg/ml) mixed into aqueous mountant (see Figure 4B).

### Immunohistochemistry

Sections lying beneath photothrombotic occlusions were mounted onto Fisher Superfrost Plus slides (Pittsburgh, Pennsylvania, United States) and underwent antigen retrieval in 10 mM citrate buffer (pH 6.0) heated to boiling in a microwave oven (300 s at full power). The slides were incubated overnight in one of four monoclonal antibodies in diluent with PBS and 0.2% (v/v) Triton X–100 followed by incubation with a biotinylated anti-mouse secondary antibody: (1) Hypoxyprobe antibody (90204; Chemicon); (2) MAP2 antibody (M1406; Sigma, St. Louis, Missouri, United States); (3) CD41 antibody (MAB1207; Chemicon); and (4) vimentin antibody (MAB3400; Chemicon). In all cases, bound antibody was visualized with the Vector ABC Kit (Vector Laboratories, Burlingame, California, United States) using diaminobenzadine as the chromagen. The sections were cover-slipped with Prolong mountant (Molecular Probes, Eugene, Oregon, United States).

## Supporting Information

Figure S1 Schematic of the Two-Photon Laser Scanning Microscope (TPLSM) Our realization is based on an 800-nm fs (femtosecond) laser with an integrated continuous-wave (CW) 532-nm laser for photothrombotic clotting. The CW laser, attenuated using neutral density (ND) filters, is directed onto the beam axis of the TPLSM with a dichroic mirror (600 nm–long pass, dichroic 1). An approximately 2-mm hole was etched in the coating of the TPLSM dichroic (dichroic 2) to allow transmission of the green laser beam. The 532-nm laser was aligned so it focused in the same plane as and at the center of the TPLSM image. The rat is bolted, via a metal head frame affixed to the skull, onto a two-dimensional translation stage that allows precise positioning of the rat relative to the TPLSM field of view and the CW laser focus. HWP, half-wave plate; PMT, photomultiplier tube.
(141 KB PDF).

Figure S2 Illustration of Automated Algorithm for Finding Slope of Streaks Formed by Moving RBCs in TPLSM Line-Scan Data The data are for the same vessel shown in Figure 1C, 1D, and 1E, although not from the same time point as Figure 1D.
(A) Line-scan data from an epoch in time are transformed to a square matrix with normalized axes. In the left image, an abrupt change in the slope of the streaks due to a heartbeat is indicated.
(B) The central region of the square matrix is rotated, and we search for the angle that yields horizontal streaks, as in the middle panel.
(C) Separability of line-scan data as a function of rotation angle; separability is maximal for vertical or horizontal streaks (Protocol S1). The rotation angle corresponding to horizontal streaks is chosen, yielding the RBC speed and direction, in this case: 11.9 mm/s and a flow direction of right to left.
(262 KB PDF).

Figure S3 Baseline Measurements of the Time-Averaged RBC Speed at the Center of a Vessel as a Function of the Diameter of the Vessel The data include all arterioles in this study. Arterioles measured as part of the photothrombotic (rose bengal) and MCA (filament) occlusion studies are indicated separately. The line represents a best-fit linear regression to the data, and shows a statistically significant correlation, valid for diameters between 10 and 130 μm, between speed, S, and diameter, D, given by S = (4.9 mm/s) + (124/s) × D (*p* < 0.0001).
(765 KB PDF).

Figure S4 Normalized Volume Blood Flux in Five Arterioles as a Function of Time For each vessel, each measurement of the flux was normalized to the average over all measurements for that vessel. The five vessels varied substantially in their average volume flux: 0.17 (circles), 0.013 (diamonds), 0.0048 (squares), and 0.0021 μl/s (triangles). The inset shows the histogram of the normalized flux for all vessels, after the long-term decrease in flux (~5% per h) is removed.
(710 KB PDF).

Figure S5 Epi-Fluorescence Images Overlaid on Wide-Field Images Showing Vascular Retention of Circulating Fluorescein-Dextran (A) The brain surface was irradiated with 1 mW of 532-nm irradiation for 1 min after an intravenous injection of rose bengal. The laser focus was deliberately located in a region where there were no surface vessels; therefore no surface vessel was clotted. The vascular retention and parenchymal extravasagation of the fluorescein-conjugated dextran used for in vivo imaging is somewhat more extensive than that observed in Figure 3. This is likely because a surface target vessel was not present to absorb and scatter the incident laser light, leading to a higher fluence incident on the sub-surface capillaries, and increasing the extent of the photochemical damage.
(B) A surface vessel was irradiated at 1 mW for 1 min without any rose bengal present. No retention of the fluorescein-dextran is evident in the sub-surface capillaries, indicating no photochemical damage.
(2.4 MB PDF).

Figure S6 MAP2 Immunohistology of Coronal Brain Slices beneath a Photothrombotic Clot of a Surface Arteriole (Ischemic Side) and from the Corresponding Location on the Contralateral Side (A, B, E, and F) Clotted side. (C, D, G, and H) Contralateral side. The boxes in panels (A), (C), (E), and (G) indicate the locations of the images in panels (B), (D), (F), and (H), respectively. The arrows in (B) and (F) indicate cells showing minor neuropathology (single arrow indicates cork-screw dendrites; double arrow indicates shrunken cells with eccentric nuclei. Most cells in panels (B) and (F) exhibit no pathology. The example on the left is the same as that shown, in part, in Figure 3. In the example on the right, some trapped RBCs are visible in capillaries beneath the photothrombotically clotted vessel (E), although the capillaries were still flowing after clot formation, based on in vivo TPLSM.
(6.8 MB PDF).

Figure S7 Control Experiment Showing that Photothrombotic Clot Formation Requires Both Rose Bengal and Green Laser Irradiation (A) Baseline TPLSM image of same vessel shown in Figure 2. The green circle indicates the region that will be irradiated with 532-nm light.
(B) After 2 min irradiation with 0.5 mW of 532-nm laser light before intravenous injection of rose bengal. No clot formation is evident.
(C) After an additional 2-min irradiation after intravenous injection of rose bengal. Forming clot indicated by green arrow.
(386 KB PDF).

Protocol S1 TPLSM for Photothrombotic Clotting and Characterization of RBC Flux A locally designed two-photon laser scanning microscope was modified to allow real-time photothrombotic clotting of individual cortical surface blood vessels. TPLSM line-scans were used to collect space–time data of RBC motion in individual cortical blood vessels, and an algorithm based on singular value decomposition was used to find the slope of the streaks formed by moving RBCs in this space–time data, thereby determining the RBC speed. Control experiments established that systematic flux changes in vessels due to intravenous injections or to shedding of clot material during the formation of a localized photothrombotic clot are less than 10%.
(419 KB PDF).

Video S1 Two-Photon Fluorescence Image Sequence Showing the Formation of a Localized Photothrombotic Clot in a Surface Arteriole This is the same example shown in Figure 2. Images are displayed at a rate that is sped up by a factor of 15. The fluctuating, streaked appearance of the vessels is due to the motion of RBCs, and indicates flow. The initial direction of flow in the targeted arteriole (vertically centered in image) is right to left, branching into three vessels on the left of the frame. A saturated white strip at the top of the frame indicates irradiation with 523-nm light. Clot material is formed just downstream from the irradiated region of the vessel, and some sheds off and is carried away down one of the downstream branches. Note that after a complete occlusion is formed, the streaked appearance is maintained in the downstream branches, indicating they are still flowing.
(7.3 MB MOV).

## Abbreviations

MAP2: microtubule associated protein 2
MCA: middle cerebral artery
NA: numerical aperture
RBC: red blood cell
TPLSM: two-photon laser scanning microscopy
