# Supplementary material for: Two-Photon Imaging of Cortical Surface Microvessels Reveals a Robust Redistribution in Blood Flow after Vascular Occlusion
Source: PLoS Biol. 2006 Jan 3;4(2):e22. doi: 10.1371/journal.pbio.0040022 (PMC1324794; doi:10.1371/journal.pbio.0040022)
Supplement: Supplementary file 1 [file pbio.0040022.sd001.pdf]

Protocol S1: TPLSM for photothrombotic clotting and characterization of RBC flux.

Supporting Information for "Two-photon imaging of cortical surface microvessels reveals a robust redistribution in blood flow after vascular occlusion" by Chris B. Schaffer, Beth Friedman, Nozomi Nishimura, Lee F. Schroeder, Philbert S. Tsai, Ford F. Ebner, Patrick D. Lyden and David Kleinfeld

Modified two-photon laser scanning microscope.

We use a locally-designed TPLSM for the studies described here that has been modified to allow delivery of green laser light for photothrombotic clotting (Fig. S1). Tightly-focused 100-fs, 800-nm pulses from the femtosecond laser oscillator (Mira 900-F, Coherent, Inc.) are scanned throughout the brain of the rat using galvanometric scan mirrors and axial translation of the objective. The energy per pulse of the imaging beam was adjusted by passing the beam through a rotating half-wavelength ($\lambda/2$) plate and keeping only one polarization with a polarizing beam splitter. Two-photon excited fluorescence was separated by a dichroic mirror (600-nm long pass; dichroic 2 in Fig. S1), spectrally filtered with BG-39 colored glass to block residual 800-nm light, and detected on a photomultiplier tube (PMT) (no. R6357; Hamamatsu). The PMT signal was amplified (10^5 transimpedance gain), low-pass filtered (4 pole Bessel at $f_c = 300$ kHz), and digitized. LabView™-based software of local design controlled the scan mirrors and microscope objective stepper motor and recorded the PMT signal as a function of the scan mirror and objective position. Using the metal head frame that is affixed to the rat's skull, the rat is bolted onto a two-dimensional translation stage underneath the microscope objective, allowing precise positioning of the rat relative to the TPLSM field of view. Individual planar images that spanned the area surrounding the target vessel, or sets of images at 1-µm axial spacing, were obtained both before and after photodisruption. Planar images were also used to monitor the production of the vascular disruptions in real-time.

A continuous wave (CW) 532-nm wavelength laser (TIM-622, Transverse Industries), which was attenuated using neutral density (ND) filters, was directed onto the beam axis of the TPLSM

with a dichroic mirror (600-nm long pass, dichroic 1 in Fig. S1). An ~ 2-mm hole was etched in the coating of the TPLSM dichroic (dichroic 2 in Fig. S1) to allow transmission of the green laser beam. This small hole resulted in no noticeable degradation of the TPLSM image. The 532-nm laser was aligned so it focused in the same plane as and at the center of the TPLSM image.

Line-scan. A key requirement to accurately determine the blood flow speed with TPLSM in line-scan mode is that the same RBC must be captured by several successive line-scans. This requires that the line-scan be along the axis of the vessel, that there be no RBC motion perpendicular to the axis of the vessel, and that the scan speed be sufficiently fast. Our apparatus allows the line-scan axis to be rotated so it lies along any direction parallel to the cortical surface, but, because axial translation of the objective is slow, we cannot tip the line-scan out of the plane of the cortical surface. Thus we are limited to measurements of vessels that lie parallel to the cortical surface. The smooth, nearly-parabolic flow profile we observe (Fig. 1f) indicates non-turbulent flow, ensuring that RBC motion is only along the vessel axis. The relatively slow variation of the RBC speed with position perpendicular to the axis of the vessel also implies that the variability in our measurement of the speed due to slight misalignment of the line-scan axis from the vessel center is minimal. Finally, we typically used a line-scan rate of 1.3 kHz, fast enough to measure flow speed in most surface microvessels.

Singular value decomposition to extract slope of RBC streaks in line-scan data

The determination of RBC speed from the streaks in the space-time line-scan data (Fig. 1d), has been automated. In brief, we parse the data into short epochs of time, and within each epoch we rotate the space-time image so that the streaks formed by the moving RBCs are horizontal (Fig. S2). The rotation necessary to meet this condition gives the slope of the streaks, which is related to the direction and average speed of the RBCs. This method correlates the data across all measured points and thus is an improvement over a two-point correlation.

Identification of the rotation that yields horizontal RBC streaks is done using singular value decomposition. Formally, we note that the line-scan data forms a two dimensional array, denoted $I(x,t)$. We pick consecutive subsets of the data over an interval τ, typically 55 ms (data in text

figure 1e used 30 ms), so that time runs from $t_0 < t < t_0 + \tau$, where t_0 is the offset into the data set. Distance runs from $0 < x < X$, where X is the length of the line-scan along the vessel and typically varies from 30 to 150 μm. The temporal average is then subtracted from the data at each position, *i.e.*, $I'(x,t) = I(x,t) - \langle I(x,t) \rangle_t$. Subtracting the average eliminates any vertical streaks in the image due to stationary features in the line-scan data, leaving only streaks formed by moving RBCs. We then rewrite $I'(x,t)$ in terms of normalized coordinates, $a = x/X - 1/2$ and $b = t/\tau - 1/2$, and interpolate the data to form a square matrix, $I'(a,b)$, with both a and b running from $-1/2$ to $1/2$. We take a square region with dimension $1/\sqrt{2}$ from the center of this data, and rotate the coordinates by an angle θ, so that $a' = a\cos\theta + b\sin\theta$, and $b' = b\cos\theta - a\sin\theta$. A reduced matrix size is chosen so that, at any rotation angle, there are no gaps in the data at the corners of the matrix. We then search for a value of θ for which the streaks in this rotated matrix, $I'(a',b')$, are horizontal. This condition is met by finding the value of θ , between –π/2 and π/2, that maximizes the separability of $I'(a',b')$. This calculation proceeds by expressing $I'(a',b')$ in terms of its singular values, $I'(a',b') = \sum_{n=1}^{N} \lambda_n F_n(a') G_n^T(b')$, where F_n and G_n are orthonormal functions of the a and b variables, respectively, λ_n is the expansion coefficient or eigenvalue, and N is the rank of $I'(a',b')$. The separability, defined as $\lambda_1^2 \Big/ \sum_{n=1}^{N} \lambda_n^2$ $\lambda_1{}^2$, is a maximum for the choice of $\theta = \theta_{\max}$ that maximizes the power in the $n = 1^{st}$ mode, so that $I'(a',b') \approx \lambda_1 F_1(a') G_1^T(b')$ (Fig. S2). A maximum separability graphically corresponds to either horizontal or vertical streaks. We manually select the $\theta_{\max}$ that corresponds to horizontal streaks, and the speed is then given by $s = X / (\tau \tan|\theta_{\max}|)$, while the direction is determined by the sign of $\theta_{\max}$.

Flux measurement controls

A critical question for the measurements presented here is the reliability of our technique for determining the blood flux (main text, with $F = (\pi/6) s d^2$ where *F* is the volume flux, *s* is the mean RBC speed and *d* is the vessel diameter), and the stability of the blood flux over the course

of hours. To address these issues we performed control experiments to estimate the systematic and random errors in our technique for determining flux.

Random errors. We repeatedly measured the diameter and speed in five different vessels in one rat, moving from one vessel to the next, over the course of about three hours. We then computed the volume blood flux. Comparing each measurement of a vessel to the average for that vessel over all measurements shows, on average, a slow decrease in blood flux over time of about 5 % per hour (Fig. S4). Based on the repeatability of the measurement, after correcting for this long-term drift, we estimate that our technique allows us to characterize the absolute volume flux in a vessel to about 10 % (inset to Fig. S4).

Systematic errors. In control experiments (three injections in three rats), we noted an approximately 10 % increase in volume blood flux immediately after injection of the rose bengal (0.3 mL, 1 % (w/v) in isotonic saline), placing a small bias on the post-clot blood flow measurements. This is an upper limit on the error because the flow likely returns to baseline values within several minutes as a consequence of homeostatic regulation mechanisms. Because the lifetime of the rose bengal in the blood stream is only about 30 min., it was not possible to perform baseline blood flow measurements after injecting rose bengal and still have a sufficiently high concentration of rose bengal after the baseline measurements for effective clotting.

During the formation of a photothrombotic clot, we typically observed chunks of clot material break away from the forming clot and get swept downstream (Supporting Video 1). This "clot shedding" could potentially form an embolus that lodges downstream from the target vessel, thereby decreasing flow and biasing our post-clot flow measurements. In control experiments (three examples in three rats), we induced the typical amount of this clot shedding we see when clotting a vessel, but without permanently occluding the vessel. Measurements of the blood flow at baseline and after this clot-shedding showed that the volume blood flux was not affected, implying the clot shedding has little effect on the flow dynamics near the target vessel.
