# Supplementary material for: Two-Photon Imaging of Cortical Surface Microvessels Reveals a Robust Redistribution in Blood Flow after Vascular Occlusion
Source: PLoS Biol. 2006 Jan 3;4(2):e22. doi: 10.1371/journal.pbio.0040022 (PMC1324794; doi:10.1371/journal.pbio.0040022)
Supplement: Supplementary file 2 [file pbio.0040022.sg001.pdf]

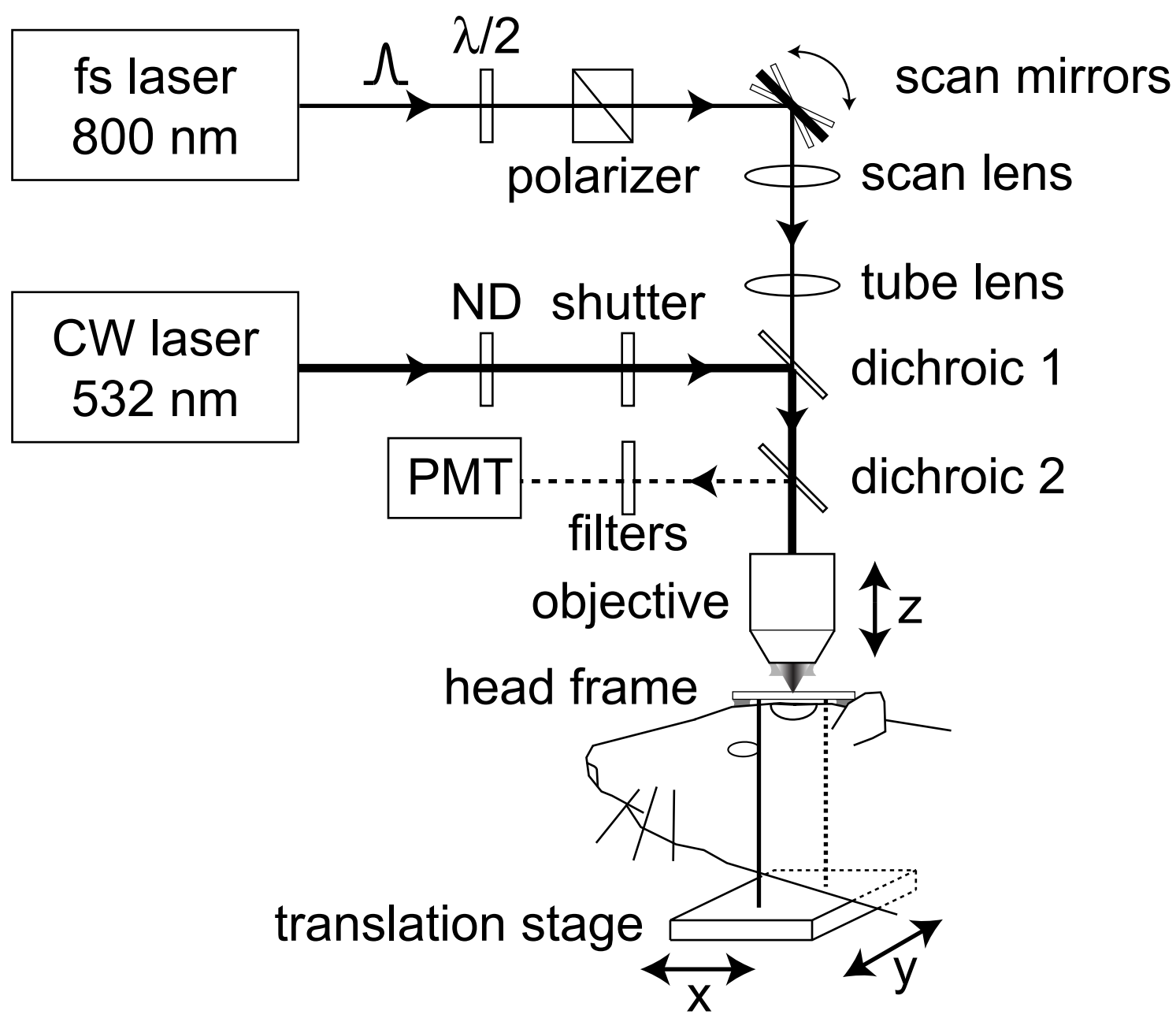

Supplementary Figure 1. Schematic of the two-photon laser scanning microscope (TPLSM). Our realization is based on an 800-nm femtosecond (fs) laser with an integrated continuous-wave (CW) 532-nm laser for photothrombotic clotting. The CW laser, attenuated using neutral density (ND) filters, is directed onto the beam axis of the TPLSM with a dichroic mirror (600-nm long pass, dichroic 1). An approximately 2-mm hole was etched in the coating of the TPLSM dichroic (dichroic 2) to allow transmission of the green laser beam. The 532-nm laser was aligned so it focused in the same plane as and at the center of the TPLSM image. The rat is bolted, via a metal head frame affixed to the skull, onto a two-dimensional translation stage that allows precise positioning of the rat relative to the TPLSM field of view and the CW laser focus. $\lambda/2$: half-wave plate, PMT: photomultiplier tube.

Schaffer, Friedman, Nishimura, Schroeder, Tsai, Ebner, Lyden and Kleinfeld
