# Supplementary material for: Two-Photon Imaging of Cortical Surface Microvessels Reveals a Robust Redistribution in Blood Flow after Vascular Occlusion
Source: PLoS Biol. 2006 Jan 3;4(2):e22. doi: 10.1371/journal.pbio.0040022 (PMC1324794; doi:10.1371/journal.pbio.0040022)
Supplement: Supplementary file 3 [file pbio.0040022.sg002.pdf]

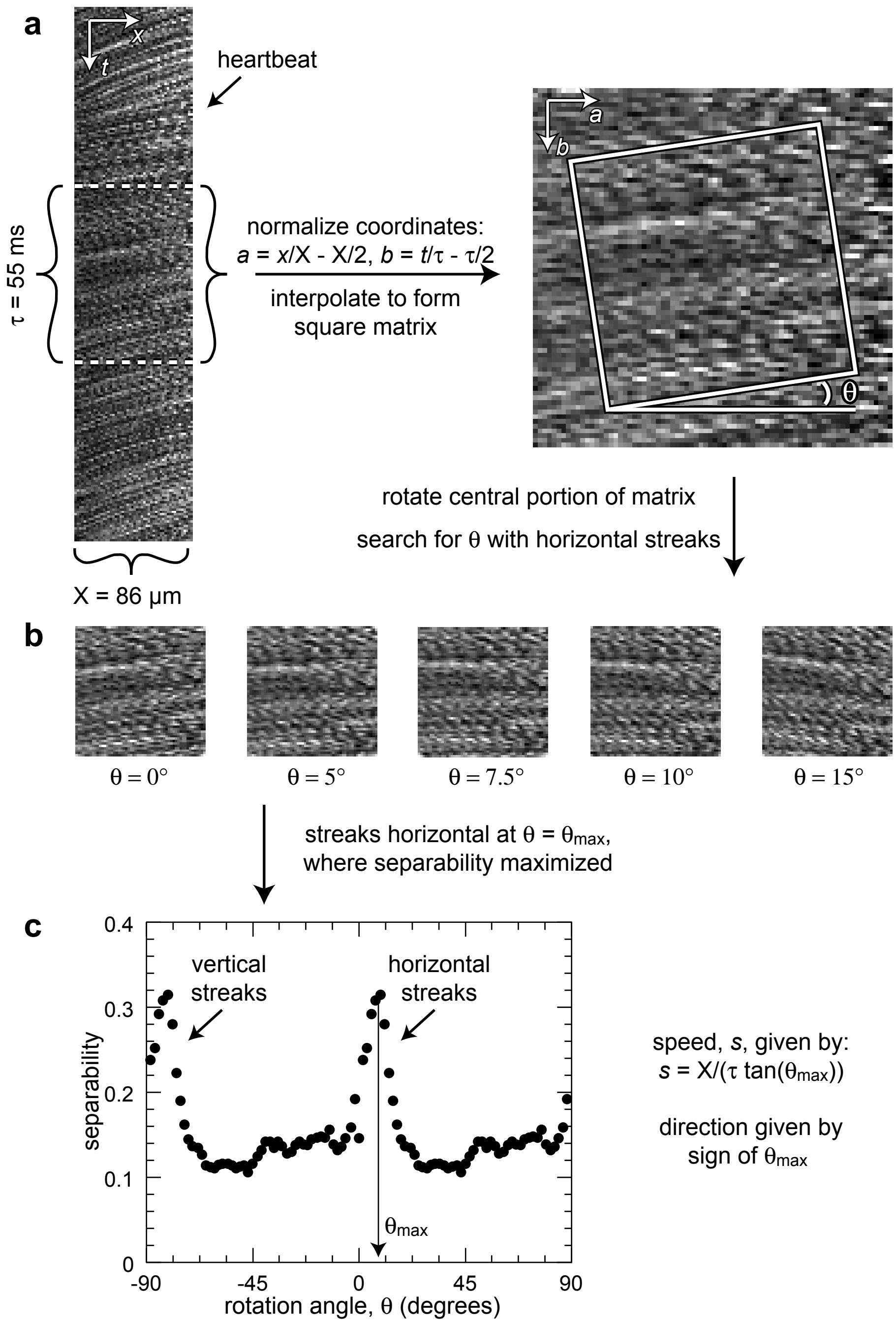

Supplementary Figure 2. Illustration of automated algorithm for finding slope of streaks formed by moving RBCs in TPLSM line-scan data. The data is for the same vessel shown in figure 1c, d, and e, although not from the same time point as figure 1d. **(a)** Line-scan data from an epoch in time is transformed to a square matrix with normalized axes. In the left image, an abrupt change in the slope of the streaks due to a heartbeat is indicated. **(b)** The central region of the square matrix is rotated, and we search for the angle that yields horizontal streaks, as in the middle panel. **(c)** Separability of line-scan data as a function of rotation angle; separability is maximal for vertical or horizontal streaks (Supplemental Text). The rotation angle corresponding to horizontal streaks is chosen, yielding the RBC speed and direction, in this case: 11.9 mm/s and a flow direction of right to left.

Schaffer, Friedman, Nishimura, Schroeder, Tsai, Ebner, Lyden and Kleinfeld
