# Supplementary material for: Two-Photon Imaging of Cortical Surface Microvessels Reveals a Robust Redistribution in Blood Flow after Vascular Occlusion
Source: PLoS Biol. 2006 Jan 3;4(2):e22. doi: 10.1371/journal.pbio.0040022 (PMC1324794; doi:10.1371/journal.pbio.0040022)
Supplement: Supplementary file 4 [file pbio.0040022.sg003.pdf]

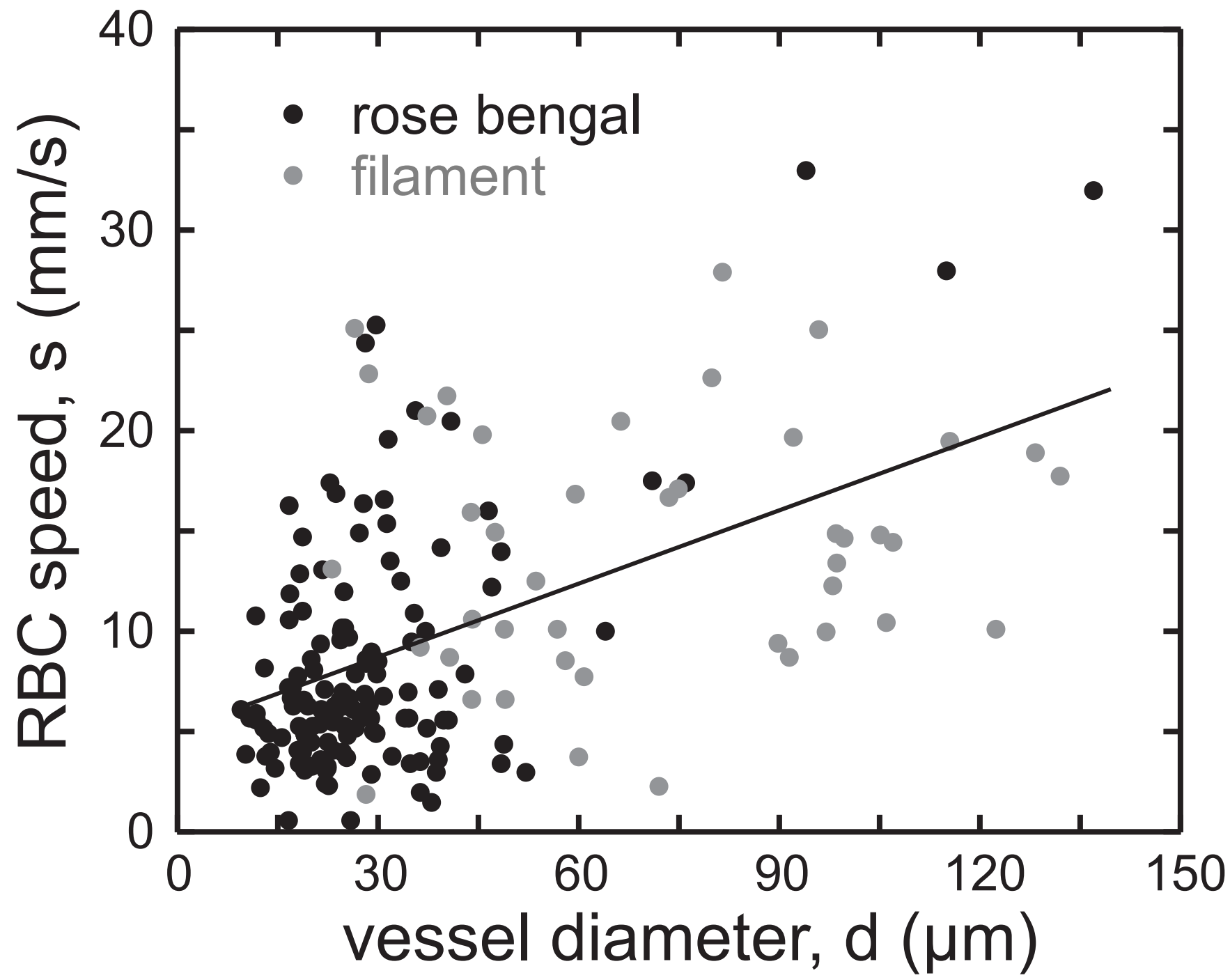

Supplementary Figure 3. Baseline measurements of the time-averaged RBC speed at the center of a vessel as a function of the diameter of the vessel. The data includes all arterioles in this study. Arterioles measured as part of the photothrombotic (rose bengal) and MCA (filament) occlusion studies are indicated separately. The line represents a best-fit linear regression to the data, and shows a statistically significant correlation, valid for diameters between 10 and 130 µm, between speed, *s*, and diameter, *d*, given by $s = (4.9 \text{ mm s}^{-1}) + (124 \text{ s}^{-1})\, d$ ($p < 0.0001$).

Schaffer, Friedman, Nishimura, Schroeder, Tsai, Ebner, Lyden and Kleinfeld
