# Supplementary material for: Two-Photon Imaging of Cortical Surface Microvessels Reveals a Robust Redistribution in Blood Flow after Vascular Occlusion
Source: PLoS Biol. 2006 Jan 3;4(2):e22. doi: 10.1371/journal.pbio.0040022 (PMC1324794; doi:10.1371/journal.pbio.0040022)
Supplement: Supplementary file 5 [file pbio.0040022.sg004.pdf]

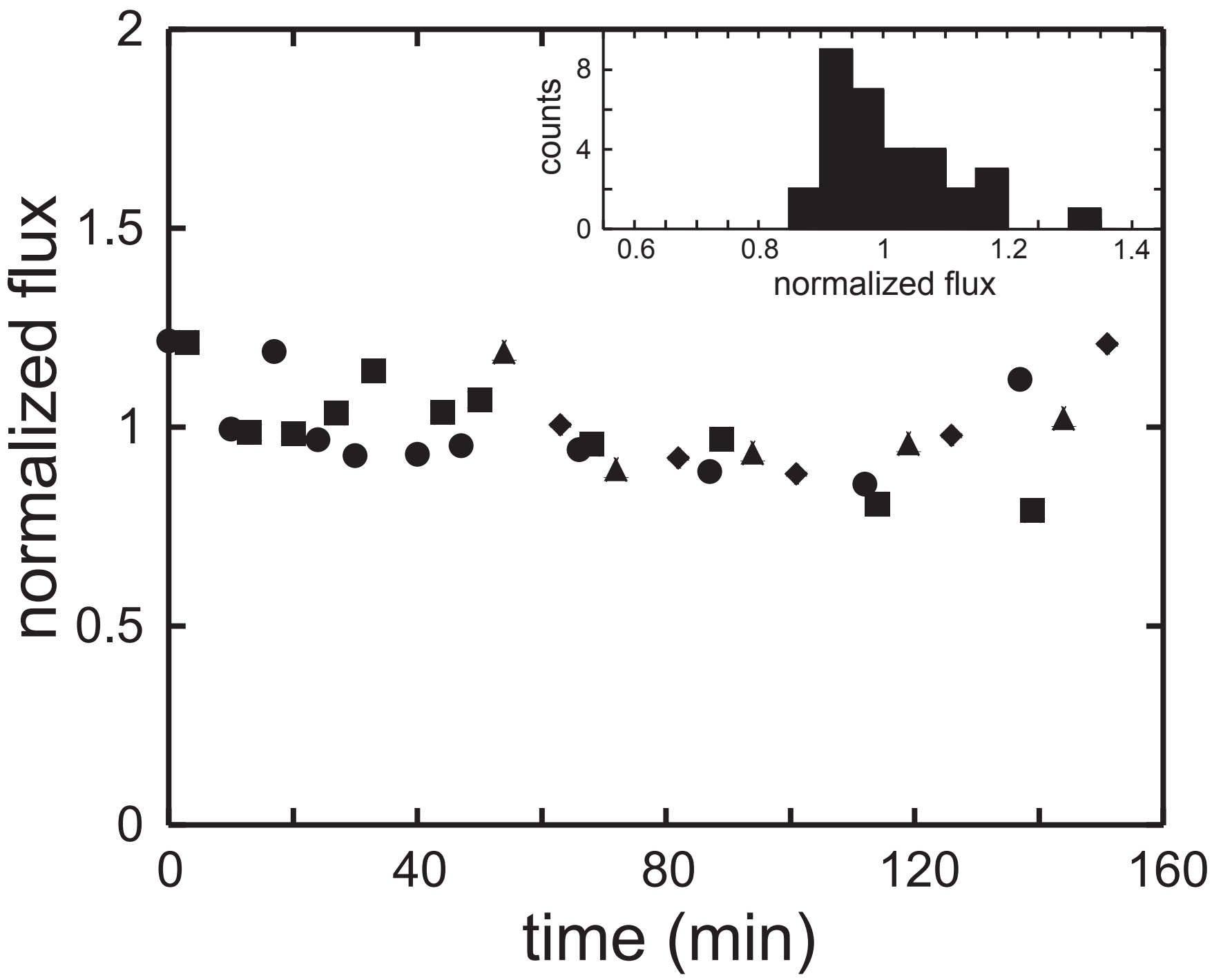


**Supplementary Figure 4. Normalized volume blood flux in five arterioles as a function of time.** For each vessel, each measurement of the flux was normalized to the average over all measurements for that vessel. The five vessels varied substantially in their average volume flux: 0.17 (circles), 0.013 (diamonds), 0.0048 (squares), and 0.0021 µL/s (triangles). The inset shows the histogram of the normalized flux for all vessels, after the long-term decrease in flux (~ 5% per hour) is removed.
