# Supplementary material for: Two-Photon Imaging of Cortical Surface Microvessels Reveals a Robust Redistribution in Blood Flow after Vascular Occlusion
Source: PLoS Biol. 2006 Jan 3;4(2):e22. doi: 10.1371/journal.pbio.0040022 (PMC1324794; doi:10.1371/journal.pbio.0040022)
Supplement: Supplementary file 6 [file pbio.0040022.sg005.pdf]

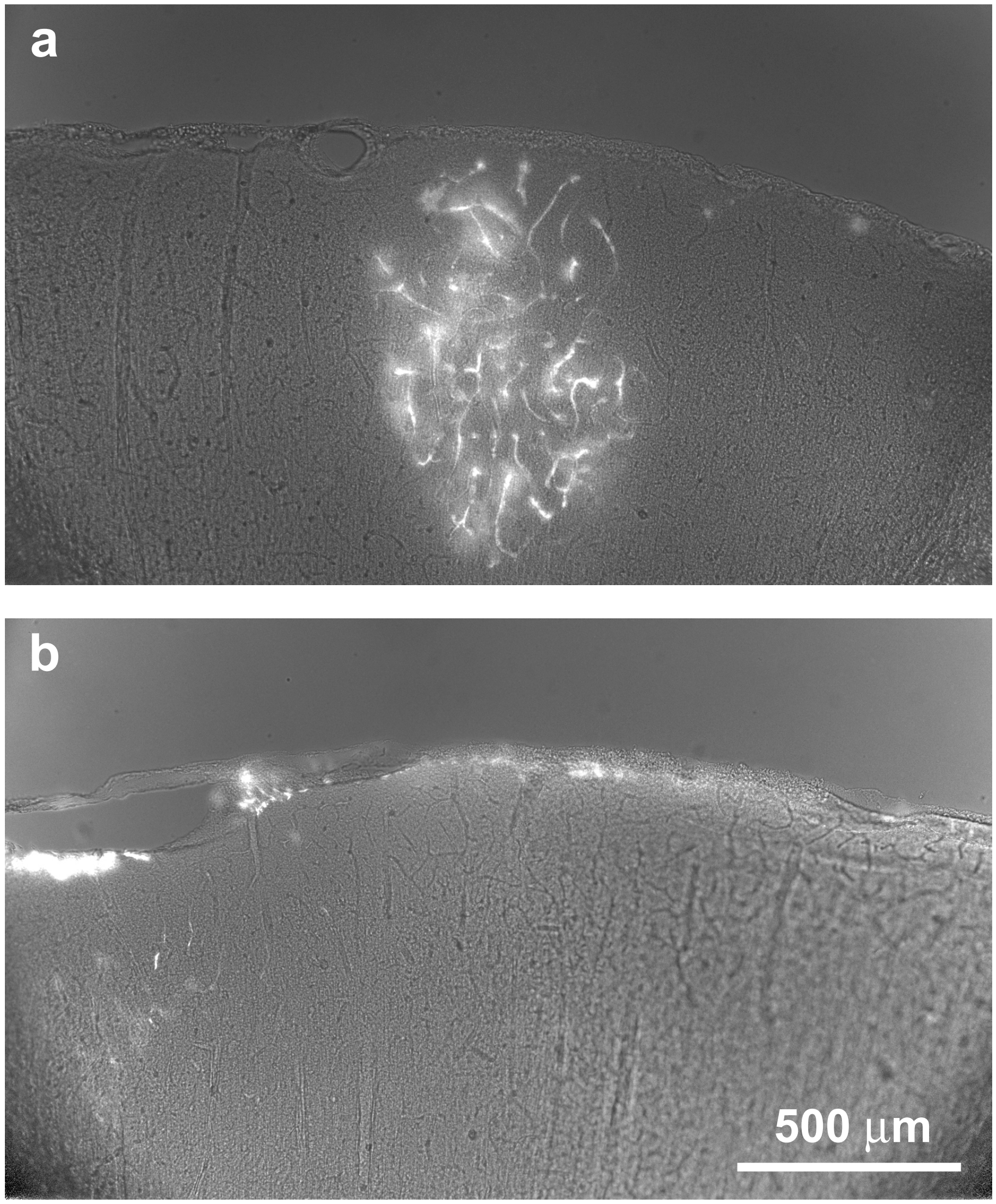

Supplementary Figure 5. Epi-fluorescence images overlaid on wide-field images showing vascular retention of circulating fluorescein-dextran. a) The brain surface was irradiated with 1 mW of 532-nm irradiation for 1 min. after an i.v. injection of rose bengal. The laser focus was deliberately located in a region where there was no surface vessels, therefore no surface vessel was clotted. The vascular retention and parenchymal extravasagation of the fluoresecin-conjugated dextran used for in vivo imaging is somewhat more extensive than that observed in text figure 3. This is likely because a surface target vessel was not present to absorb and scatter the incident laesr light, leading to a higher fluence incident on the sub-surface capillaries, and increaseing the extent of the photochemical damage. **b)** A surface vessel was irradiated at 1 mW for 1 min. without any rose bengal present. No retention of the fluroescein-dextran is evident in the sub-surface capillaries, indicating no photochemical damage.
