# Supplementary material for: Two-Photon Imaging of Cortical Surface Microvessels Reveals a Robust Redistribution in Blood Flow after Vascular Occlusion
Source: PLoS Biol. 2006 Jan 3;4(2):e22. doi: 10.1371/journal.pbio.0040022 (PMC1324794; doi:10.1371/journal.pbio.0040022)
Supplement: Supplementary file 7 [file pbio.0040022.sg006.pdf]

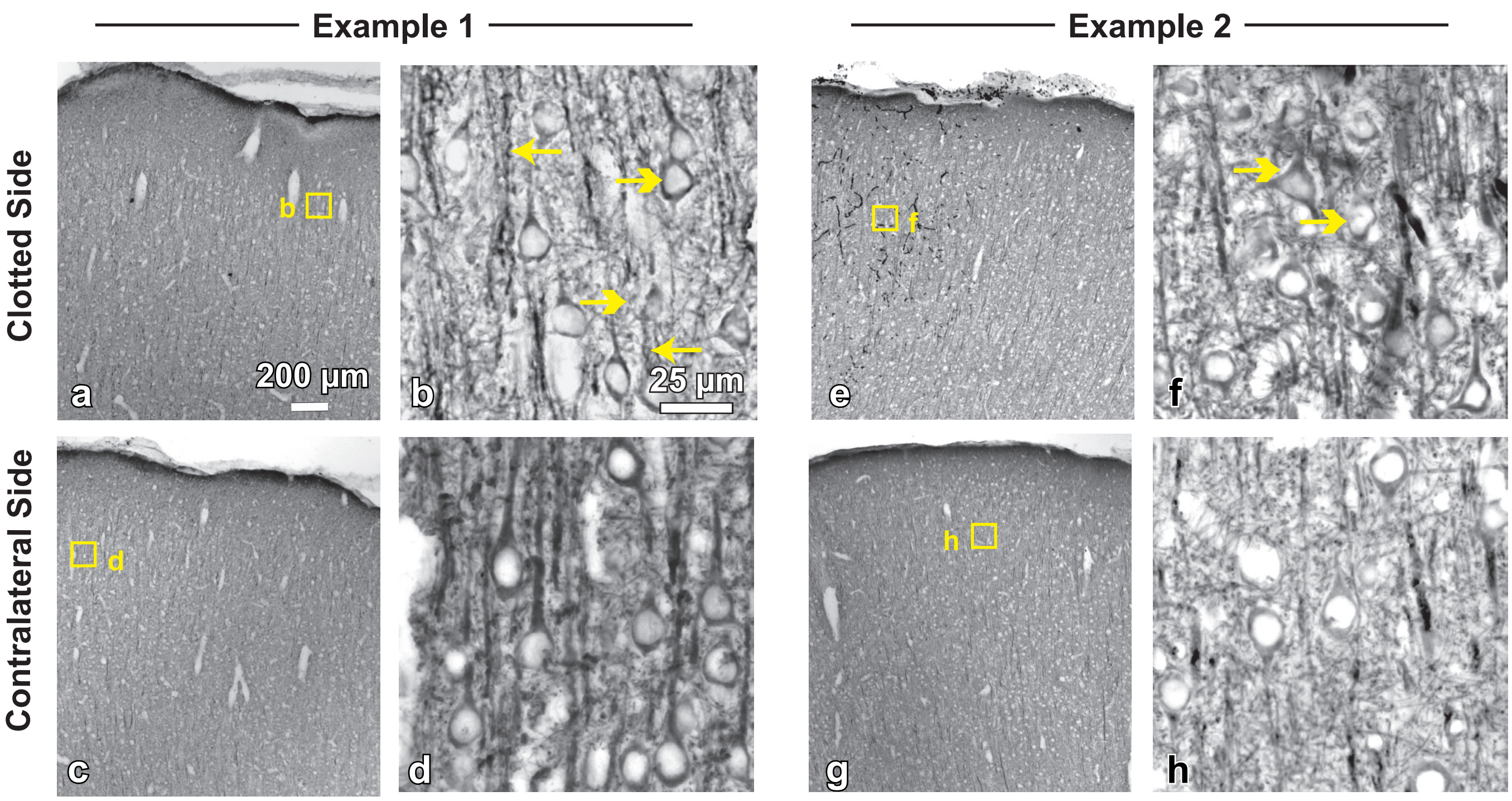

Supplementary Figure 6. MAP2 immunohistology of coronal brain slices underneath a photothrombotic clot of a surface arteriole (ischemic side) and from the corresponding location on the contralateral side. (a, b, e, and f) Clotted side. **(c, d, g, and h)** Contraleteral side. The boxes in panels (a), (c), (e), and (g) indicate the locations of the images in panels (b), (d), (f), and (h), respectively. The arrows in (b) and (f) indicate cells showing minor neuropathology (single arrow, cork-screw dendrites; double arrow, shrunken cells with eccentric nuclei. Most cells in panels (b) and (f) exhibit no pathology. The example on the left is the same as that shown, in part, in figure 3 in the text. In the example on the right, some trapped red blood cells are visible in capillaries underneath the photothrombotically clotted vessel (panel (e)), although the capillaries were still flowing after clot formation, based on *in vivo* TPLSM.
