# Supplementary material for: Two-Photon Imaging of Cortical Surface Microvessels Reveals a Robust Redistribution in Blood Flow after Vascular Occlusion
Source: PLoS Biol. 2006 Jan 3;4(2):e22. doi: 10.1371/journal.pbio.0040022 (PMC1324794; doi:10.1371/journal.pbio.0040022)
Supplement: Supplementary file 8 [file pbio.0040022.sg007.pdf]

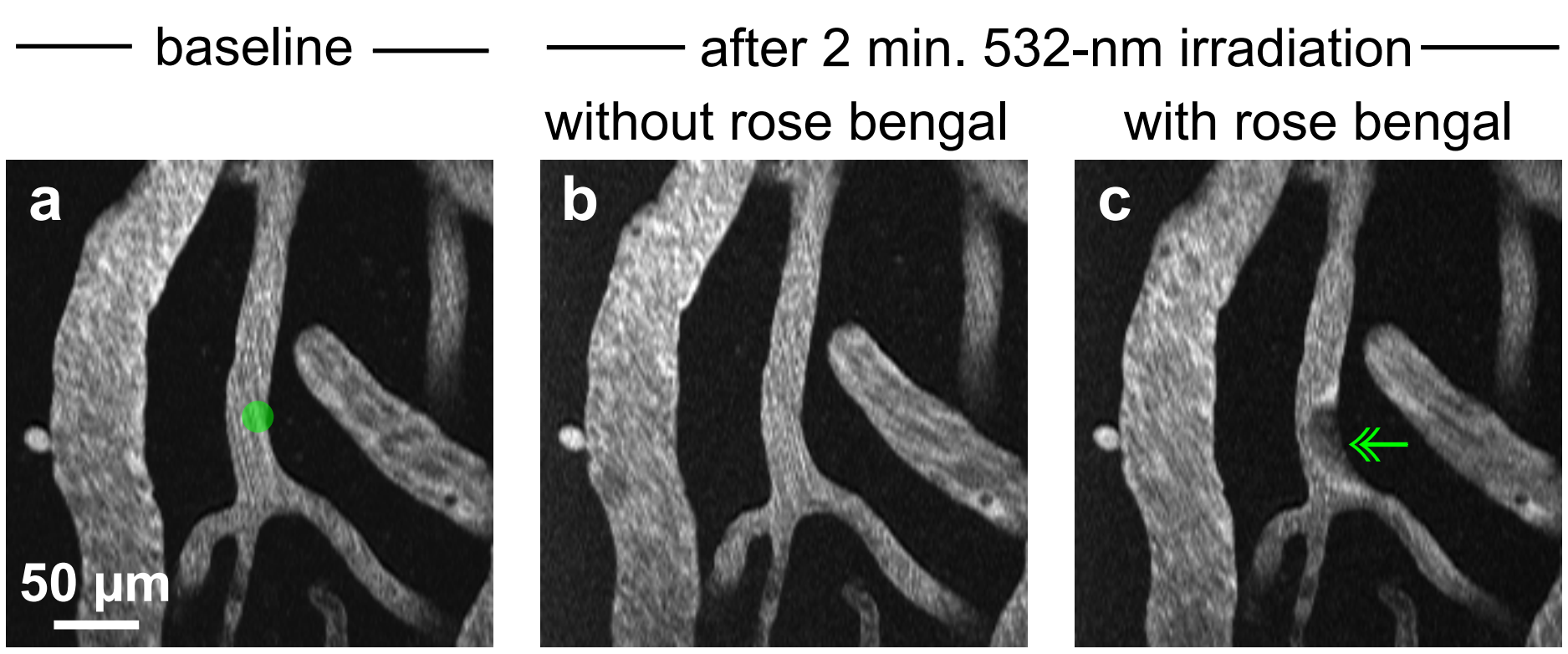

Supplementary Figure 7. Control experiment showing that photothrombotic clot formation requires both rose bengal and green laser irradiation. (a) Baseline TPLSM image of same vessel shown in figure 2. The green circle indicates the region that will be irradiated with 532-nm light. **(b)** After 2 min. irradiation with 0.5 mW of 532-nm laser light before intravenous injection of rose bengal. No clot formation is evident. **(c)** After an additional 2 min. irradiation after intravenous injection of rose bengal. Forming clot indicated by green arrow.
